# Western classical music development: a statistical analysis of composers similarity, differentiation and evolution

**DOI:** 10.1007/s11192-017-2387-x

**Published:** 2017-04-22

**Authors:** Patrick Georges

**Affiliations:** Graduate School of Public and International Affairs, University of Ottawa, Social Sciences Building, Room 6011, 120 University, Ottawa, ON K1N 6N5 Canada

**Keywords:** Classical composers, Influences network, Similarity indices, Imitation, Differentiation, Evolution

## Abstract

This paper proposes a statistical analysis that captures similarities and differences between classical music composers with the eventual aim to understand why particular composers ‘sound’ different even if their ‘lineages’ (influences network) are similar or why they ‘sound’ alike if their ‘lineages’ are different. In order to do this we use statistical methods and measures of association or similarity (based on presence/absence of traits such as specific ‘ecological’ characteristics and personal musical influences) that have been developed in biosystematics, scientometrics, and bibliographic coupling. This paper also represents a first step towards a more ambitious goal of developing an evolutionary model of Western classical music.

## Introduction

This paper has two objectives. First, the paper contributes to the music information retrieval literature by establishing similarities between classical music composers.^1^ That two composers, or their music, ‘sound alike’ or ‘sound different’ is inherently a subjective statement, made by a listener, which depends on many factors, including the degree of familiarity to classical music per se.^2^ This paper addresses the subjective issue, using well-established similarity indices (e.g., the centralised cosine similarity measure) based on measurable criteria. Even if no audio file is used in the analysis, ‘sounding alike’ is used in this paper as a proxy (or shortcut) with the specific meaning that the music of two composers is similar in ecological/musical characteristics and/or personal musical influences (as defined below). Uncovering what makes two composers similar, in a systematic way, has important economic implications for (1) the music information retrieval business; (2) a deeper insight into musical product definition and choice offered to music consumers and purchasers and (3) for our understanding of innovation in the creative industry.

**Fig. 1:**
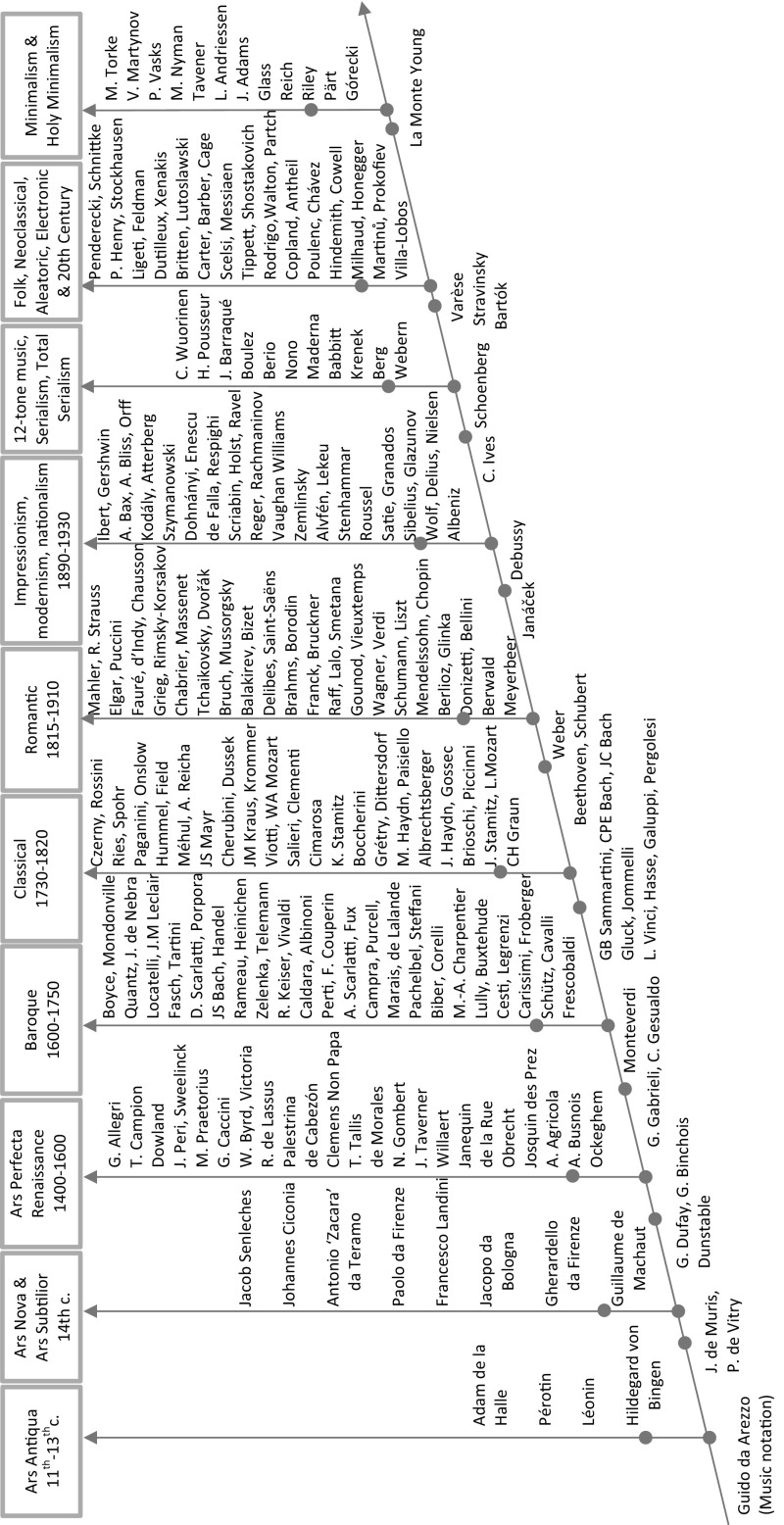
Partial outline of Western classical music and composers. *Note* At the bottom of vertical lines we find ‘earlier’ composers (e.g., Frescobaldi for early Baroque); at the top we find ‘later’ composers (e.g., JS Bach for of High/Late Baroque). Composers located along vertical lines have pursued and developed further the style of their periods with some degree of intra-period cross-imitation. Along the diagonal line we find ‘transitional’ composers and/or ‘innovators. According to music historians (some of) their works have contributed to a transition from one style/period to another. *Source*: Assembled by the author on the basis of general music information and dictionaries (e.g., Taruskin and Gibbs 2013)

This leads to a second objective of the paper, which is to propose a statistical framework that could identify transitional figures, innovators and followers in the development of Western classical music. Western classical music evolved gradually, branching out over time and throwing off many new styles. This overall development is not due to simple creative genius alone, but to the influence of past masters and genres, as constrained or facilitated by the cultural conditions of time and place. Figure 1 conveys this development and proposes a (narrow) historical time line for music periods (e.g., Medieval, Renaissance, Baroque, Classical, Romantic and Modern/twentieth century) and some composers belonging to these periods.^3^ Along vertical lines are composers who have developed and perfected (or pushed to the limit) the musical style of their period. Others composers (not necessarily shown), may gravitate around them, extending the volume of music production in an essentially imitative style. Along the diagonal line are some ‘transitional’ and/or ‘innovative’ composers whose works (or at least some of them) have been assessed by musicologists to contribute to a transition from one period to another.^4^

As claimed by Gatherer (1997), “a dialectical approach to music evolution would seek to identify the internal stylistic tensions and contradictions (in terms of thesis and antithesis) which give rise to new musical forms (synthesis).” Franz Brendel (1811–68), a doctor of philosophy, is the first self-consciously Hegelian historian of music and, according to Taruskin and Gibbs (2013), henceforth T&G (2013), his great achievement was to write the nineteenth century’s most widely disseminated history of music.^5^ Brendel casts his narrative in terms of successive emancipations of composers and the art of music (emancipation from the sacred, emancipation from words, etc.). For T&G (2013), through this Hegelian approach, “many people have believed that the history of music has a purpose and that the primary obligation of musicians is not to meet the needs of their immediate audience, but, rather, to help fulfill that purpose—namely, the furthering of the evolutionary progress of the art. This means that one is morally bound to serve the impersonal aims of history, an idea that has been one of the most powerful motivating forces and one of the most demanding criteria of value in the history of music. (…). With this development came the related views that the future of the arts was visible to a select few and that the opinion of others did not matter.” This Hegelian perspective claims to show why things changed. This makes it fundamentally different from Darwin’s theory of biological evolution based on random mutation. Change or evolution in the Hegelian approach is viewed as having a purpose, which turns random process into a law.

For Gatherer (1997), “a Darwinian alternative to dialectics, which in its most reductionist form is known as memetics, seeks to interpret the evolution of music by examining the adaptiveness of its various component parts in the selective environment of culture.” The diagonal in Fig. 1 (and the identified composers along the diagonal) could represent a somewhat lengthy process of music ‘speciation’ so to speak (in analogy to evolutionary biology).^6^ Darwinian biological models have been applied to many aspects of cultural evolution (see Linquist 2010 for one good survey), but not so much to music (see, however, Gatherer 1997; Jan 2007). An evolutionary approach to classical music could perhaps be narrated along the following lines. Music transmission is analogous to genetic transmission in that it can give rise to a form of evolution by selection. By planting a fertile ‘meme’ in another composer mind, the initial composer manipulates his brain, turning it into a vehicle for the meme’s propagation.^7^ Composition imitation is how musical memes can replicate. However, the inherited music style adapts to local ecological and social conditions by a process of musical mutation/variation and differential fitness that is akin to natural selection.^8^ Just as not all genes that can replicate do so successively, so some music memes are more successful in the meme-pool than others, leading to a process of ‘musical’ (instead of natural) selection, a non-random survival of random musical mutations. In other terms, musical memes are passed on in an altered form, through musical mutation and speciation, branching out over time into many new and diverse styles.

This suggested, the present paper does not go deeply into any ‘pseudo-scientific’ meta-narrative for Western classical music evolution. Rather, and more modestly, it proposes a statistical analysis that captures similarities and differences between classical music composers. The eventual aim is to increase our understanding of why particular composers ‘sound’ different even if their ‘lineages’ (or personal influences network) are similar, thereby contributing to an evolution in Western classical music. Musicologists and music historians have described and classified composers, the styles and the periods in which they lived. They have discussed the relationships and influences network of composers, the evolution of music styles, who they see as transitional figures, innovators, or followers. See for example the History of Western Music by T&G (2013), a History of Opera by Abbate and Parker (2012), Grout and Williams (2002) and many others. Typically, these authors use descriptive narratives and music manuscripts analyses. The objective of this paper is to complement these approaches by proposing a statistical analysis that captures similarity across pairs of composers by mean of pairwise comparison of presence-absence of traits such as personal musical influences and musical/ecological characteristics. To this end, we use an approach that is based on (but different from) the earlier contributions by Smith and Georges (2014, 2015), using methods that have been developed in biosystematics, scientometrics, and bibliographic couplings.

The rest of the paper is as follows. The first section describes the data (influences network and ecological characteristics) and the methodology used in Smith and Georges (2014, 2015). The second section shows how the interaction of personal musical influences and ecological characteristics can provide a typology that could, in theory, lead to some evolutionary model of Western classical music. The third section introduces the centralised cosine measure as a statistical measure of similarity between composers.^9^ The fourth section discusses some statistical results and the last section concludes.

## Data and background information on composers’ similarity

**Fig. 2:**
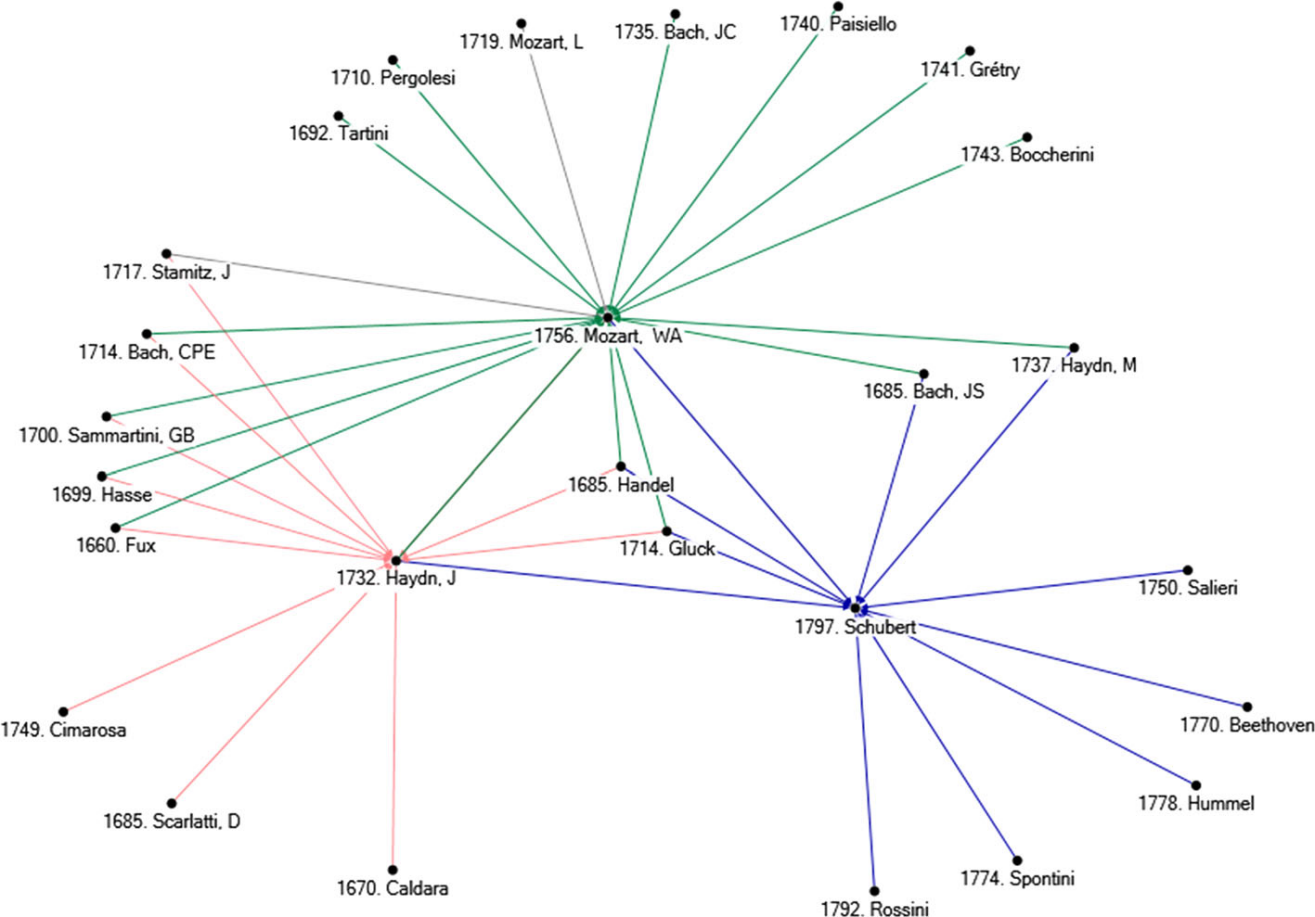
Personal musical influences on J. Haydn, W. A. Mozart, and Schubert. *Note* The *number* in front of a composer’s name in figure corresponds to his date of birth. *Source*: Constructed by the author on the basis of data collected form ‘The Classical Music Navigator’ (Smith 2000)

Smith and Georges (2014, 2015) used data collected in the ‘The Classical Music Navigator’ (Smith 2000; hereafter referred to as *CMN*).^10^ One important part of the *CMN* is the presentation of composers’ personal musical influences. Each of the 500 composers of the database is associated with a list of composers who have had a documented influence on a subject composer. Smith and Georges (2015) provide the following example in Fig. 2 which represents the network of influences on three composers, J. Haydn, W. A. Mozart, and Schubert, three Austrian composers, born respectively in 1732, 1756, and 1792, and who are typically associated with the ‘Classical’ period of Western classical music with Schubert also being a transitional composer between the Classical and Romantic periods. A casual listening to J. Haydn, W. A. Mozart, and Schubert suggests similarities across them, although to a majority of listeners J. Haydn and W. A. Mozart would probably sound ‘closer’ than J. Haydn and Schubert, or W. A. Mozart and Schubert. To overcome the subjectivity issue noted in the Introduction, Smith and Georges (2014) infer similarities among composers by assuming that if two composers share many of the same personal musical influences, their music will likely have some similarities. On the other hand, if two composers have been influenced by very distinct sets of composers, then their music is likely to have little similarity. Observe in Fig. 2 that these three subject composers share in common two particular influences: Handel and Gluck. There are no further common influences between Schubert and Haydn, but two additional common influences between Schubert and W. A. Mozart (M. Haydn and J. S. Bach) and five additional common influences between Haydn and Mozart. According to the assumption of Smith and Georges (2014), then, the larger number of common personal influences between J. Haydn and W. A. Mozart would cause (or even explain) the higher similarity between the music of these two composers than between Schubert and Mozart, let alone Schubert and J. Haydn. The third section confirms this with a methodology that generates similarity scores between any pair of composers, by means of pairwise comparison of presence-absence of personal musical influences, using the centralised cosine similarity measure.^11^

**Fig. 3:**
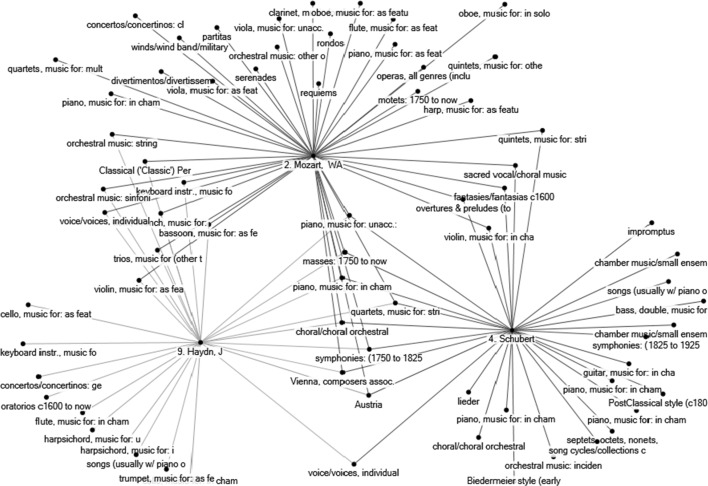
Musical ecological niches of J. Haydn, W. A. Mozart, and Schubert. *Note* The names of the ecological characteristics are truncated but their full names are given in Table 1. *Source*: Constructed by the author from raw data collected in ‘The Classical Music Navigator’ (Smith 2000), and reorganised

A second collection of data in the *CMN* associates each of the 500 composers with characteristics such as time period, geographical location, school association, instrumentation emphases, etc., and for convenience denoted ‘ecological’ categories. Smith and Georges (2015) have extracted 298 such ecological categories from the *CMN*. (See their paper for a complete list.) Thus, each composer is associated with a list of ecological categories, and the authors infer a statistical association between pairs of composers by assuming that if two composers share many ecological categories, then their musical ‘ecological niches’ are very similar, so that, in this sense, they may be considered similar. Figure 3 pursues the previous example for composers J. Haydn, W. A. Mozart, and Schubert and illustrates their musical ecological niches.^12^ We see that Mozart and J. Haydn share a larger number of ecological characteristics than, say, J. Haydn and Schubert. The contention is that this would cause a stronger similarity in the music of W. A. Mozart and J. Haydn than in the music of Schubert and J. Haydn. As before, it is also possible to compute similarity scores between any pair of composers, by means of pairwise comparison of presence-absence of ecological categories, and this will be implemented in the third section using the centralised cosine similarity measure. By introducing ecological characteristics, the basic objective in Smith and Georges (2015) was to explore the robustness of their earlier (2014) similarity results based on personal musical influences. They further propose a final list combining the ecological and influences network databases to assess similarities, arguing that this should produce a general improvement in the similarity rankings.

**Table 1:** Ecological characteristics associated with J. Haydn, W. A. Mozart, and Schubert. *Source*: Assembled from raw data collected in ‘The Classical Music Navigator’ (Smith 2000), and reorganised

Austria
Bass, double, music for
Bassoon, music for: as featured instr. w/orchestra
Biedermeier style (early nineteenth century) composers
Cello, music for: as featured instr. w/orch. (c1700–1850)
Cello, music for: in chamber music setting (c1700–1850)
Chamber music/small ensemble, general (multiple works, and for various forms): (1825–1925)
Chamber music/small ensemble, general (multiple works, and for various forms): (c1600–1825)
Choral/choral orchestral music, w/or w/o individual voice(s), general (multiple works, and for various genres) (1825–1925)
Choral/choral orchestral music, w/or w/o individual voice(s), general (multiple works, and for various genres) (c1650–1825)
Clarinet, music for: in chamber music setting (c1775–1900)
Classical (‘Classic’) Period (c1750–c1825) composers
Concertos/concertinos: clarinet c1775–now
Concertos/concertinos: general (multiple works, and for various featured instrs.) (c1700–1850)
Divertimentos/divertissements
Fantasies/fantasias c1600–now
Flute, music for: as featured instr. w/orch. c1700–now
Flute, music for: in chamber music setting c1700–now
Guitar, music for: in chamber music setting c1775–now
Harp, music for: as featured instr. w/orchestra
Harpsichord, music for: in chamber or orchestral settings c1700–now
Harpsichord, music for: unacc. c1600–c1775+
Horn, French, music for: as featured instr. w/orch. c1700–now
Impromptus
Keyboard instr., music for c1500–c1775+ : in chamber or orchestral settings
Keyboard instr., music for c1500–c1775+ : unacc.
Lieder
Masses: 1750–now
Motets: 1750–now
Oboe, music for: as featured instr. w/orch.: c1700–now
Oboe, music for: in solo or chamber music settings: c1700–now
Operas, all genres (including chamber operas): (c1600–1800)
Oratorios c1600–now
Orchestral music: incidental music to plays, etc. (and suites drawn from the latter)
Orchestral music: other orchestral forms, or general: (c1675 to1800)
Orchestral music: sinfonia concertantes and sinfonias
Orchestral music: string orchestras, music for
Overtures and preludes (to stage works)
Partitas
Piano, music for: as featured instr. w/orch. c1775–now
Piano, music for: in chamber music setting: misc. specific combinations (especially sonatas w/other instrs.) c1775–now
Piano, music for: in chamber music setting: piano four hands/two players c1775–now
Piano, music for: in chamber music setting: piano quartets c1775–now
Piano, music for: in chamber music setting: piano quintets c1775–now
Piano, music for: in chamber music setting: piano trios c1775–now
Piano, music for: unacc.: (c1775–1900)
PostClassical style (c1800–c1850)
Quartets, music for: multiple works, or for other instrumental combinations
Quartets, music for: string quartets (form or forces) c1750–now
Quintets, music for: other combinations
Quintets, music for: string quintets (form or forces)
Requiems
Rondos
Sacred vocal/choral music (various genres): (1600–1850)
Septets, octets, nonets, music for
Serenades
Song cycles/collections c1800–now
Songs (usually w/piano or orchestral accompaniment): (1800–1900)
Songs (usually w/piano or orchestral accompaniment): (c1550–1800)
Symphonies: (1750–1825)
Symphonies: (1825–1925)
Trios, music for (other than piano trios)
Trumpet, music for: as featured instr. w/orchestra
Vienna, composers assoc. w/, (c1650–1850)
Viola, music for: as featured instr. w/orchestra
Viola, music for: unacc. or in a chamber music setting
Violin, music for: as featured instr. w/orch.: (c1650–1850)
Violin, music for: in chamber music setting: (c1650–1850)
Voice/voices, individual featured, w/orchestra (contexts exclusive of opera): (1800–1900)
Voice/voices, individual featured, w/orchestra (contexts exclusive of opera): (c1625–1800)
Winds/wind band/military band music

This new paper, however, proposes a different approach. First, a new measure of similarity, equipped with a statistical significance test, the ‘centralised cosine measure’ is used, instead of the binomial index of dispersion used in Smith and Georges (2014, 2015). The centralised cosine measure is based on earlier literature in scientometrics and bibliographic couplings. Second, instead of merely *combining* together personal musical influences and ecological characteristics (to produce an improvement in similarity rankings) as proposed in Smith and Georges (2015), this paper points out that some additional information can be gained when the two sets of similarity indices are *compared*, especially when they provide conflicting information, leading to interesting questions such as why particular composers sound different (e.g., composed in different ecological niches) even if they have been influenced by the same personal musical influences and why they sound similar (e.g., composed in similar ecological niches) even in the absence of a common set of personal musical influences. The next section therefore develops a typology that highlights conflicting or reinforcing results, based on the influences network and ecological characteristics approaches, in a framework somewhat reminiscent of a biological evolutionary model.

## Music evolution: a typology based on influences networks and ecological data

Personal musical influences lead to a sort of lineage among composers. If two composers have been musically influenced by, roughly, the same list of composers, they share the same “cultural gene” pool. In this case I refer to them as ‘Most Similarly-Influenced Composers’. Because of their common personal musical influences we might expect these composers to develop a roughly similar style of music and eventually to ‘sound’ similar. However, if they do not, this should lead to hypotheses as to why a pair of composers might have very similar personal influences and yet produce very different music. Therefore, we need a second set of data to help categorise the musical style of each composer, the ecological characteristics of music referred to in the previous section. I refer to a pair of composers sharing a large set of common ecological characteristics (and thus having very similar ecological niches) as ‘Most Ecologically-Related Composers’.

Table 2 illustrates the interaction between these two dimensions. If most similarly-influenced composers (on the basis of individual musical influences) are also most ecologically-related composers (on the basis of ecological data), then those composers are most similar (they share a very similar set of personal musical influences and a very similar set of ecological characteristics, that is, very similar ecological niches). In terms of Fig. 1, these composers are likely to be grouped into one of the vertical lines of the ‘tree’. At the other extreme we have most dissimilar composers. In Fig. 1, it could be composers belonging to non-connected vertical lines representing very distinct musical periods and styles. But there are two other, perhaps more interesting, cases. First, why do composers produce music that ‘sounds’ different if they have the same lineage/personal musical influences? As mentioned in the Introduction, some composers may have developed a different music style through a process of ‘musical’ selection and ‘speciation’ whereby an inherited musical style adapts to local and social conditions through mutation/variation and differential fitness/competition that is akin to natural selection. If a subject composer is very similar to a series of other (contemporary) composers in terms of personal musical influences but at the same time mostly ecologically unrelated to them, then the music of this composer is likely to ‘sound’ different, to have evolved. In Table 2 this is represented as ‘music speciation and evolution’. In Fig. 1, this would be represented by composers along the diagonal line (e.g., Gluck, Debussy, Schoenberg, etc.). The second interesting case is why particular composers ‘sound’ alike if their lineage is different? Two composers, although perhaps geographically distant, may have composed music that sounds alike because they belong to very similar musical ecological niches that lead to selection pressures to adapt and develop similar sounding forms, despite having a very different lineage, in a process that could be called musical ‘convergent evolution’. See Table 2. In biology, one can identify convergent evolution wherein species that live in similar but geographically-distant habitats will experience similar selection pressures from their environment, causing these to evolve similar adaptations, or converge, coming to look and behave very much alike even when originating from very different lineages.^13^ However, this possibility seems less likely in the case of Western classical music because the time frame is rather short and the spatial frame is small, so that ‘convergence’ may only play a rather minor role in the overall process of musical evolution. A simpler interpretation is that a composer, having little documented personal musical influences in common with another contemporary composer, and therefore being perhaps (although not necessarily) isolated in the network of composers, has nevertheless composed in an ecological niche reminiscent of the musical style of the other composer, producing music that sounds similar. By being imitators or followers, and perhaps not central to the musical scene, these composers contributed less to the evolution of the sound of Western classical music.

**Table 2:** A typology of similarities for pairs of composers

	Similarity of personal musical influences Influences network data (personal lineage)	
	Low (*Most dissimilarly*-*influenced composers*)	High (*Most similarly*-*influenced composers*)
Similarity of musical ecological niches Ecological characteristics data		
High (*Most ecologically*-*related composers*)	Adaptation: Convergent evolution^a^	Most similar composers
Low (*Most ecologically*-*unrelated composers*)	Most dissimilar composers	Adaptation: Music speciation and evolution^b^

Figure 6a–p in the fourth section will provide a visual representation of the table for any ‘subject’ composer with respect to all other 499 composers of the *CMN*

^a^Pairs of composers sounding alike despite lack of common lineage

^b^Pairs of composers sounding different despite a common lineage

## The centralised cosine measure as an index of association/similarity

This section describes the methodology used in this article to assess the relationship (association/similarity) between pairs of composers. The discussion is couched in terms of personal musical influences but the methodology related to ecological categories is analogous. I first describe how I have conceptually organised the *CMN* database. This description draws on earlier articles by Smith and Georges (2014, 2015) and Smith et al. (2015). Suppose the set *C* of all 500 composers (*n* = 500) who are included in the *CMN*. For any pair of composers (*i*, *j*) for $i,j \in C$ (among the *n* × *n* possible pairs), we are interested in capturing whether a composer $k \in C$ had a reported influence on both *i* and *j*, on *i* but not *j*, on *j* but not *i*, and on neither *i* nor *j*. Running this across all composers *k* for each pair (*i*, *j*) we eventually obtain the set *I*_*i*_ of all personal influences on composer *i*, and the set *I*_*j*_ of all personal influences on composer *j*. Also, for any pair (*i*, *j*), $I_{i} \cap I_{j} = CI_{i,j}$ is the set of composers *k* that have influenced both *i* and *j*; $I_{i} - I_{i} \cap I_{j} = I_{i, - j}$ is the set of composers *k* that have influenced *i* but not *j*; $I_{j} - I_{i} \cap I_{j} = I_{j, - i}$ is the set of composers *k* that have influenced *j* but not *i* and $DI_{i,j} = I_{i, - j} \cup I_{j, - i}$ is the set of composers *k* that have influenced either *i* or *j* but not both. From this we can produce a count table, given in Table 3, for any pair (*i*, *j*) that sums the elements (the number of composers) in each of the four sets $CI_{i,j}$, $I_{i, - j}$, $I_{j, - i}$, and $C - CI_{i,j} - DI_{i,j}$, and from which similarity indices for all pairs of composers (*i*, *j*) can be computed on the basis of well-known formulas.^14^

**Table 3:** 2 by 2 frequency table for Presence/Absence of personal influences using counts

	Composer *j*		
	Presence	Absence	Total
Composer *i*			
Presence	*a*	*b*	*a* + *b*
Absence	*c*	*d*	*c* + *d*
Total	*a* + *c*	*b* + *d*	*n*

In what follows I focus on the ‘centralised’ cosine measure in part because (unlike many other indices) this measure can be used to judge the statistical significance of the association between two composers.^15^ Although the centralised cosine formula is based on the concepts underlying Table 3, it is not a straightforward application and therefore, it requires a slightly more structured presentation in order to establish a connection with the table. Here, the discussion follows closely Smith et al. (2015). The ordinary (non-centralised) cosine similarity measure (also known as the Salton’s measure) is a statistic familiar to bibliometrics and scientometrics. The idea was mathematically formalized by Sen and Gan (1983) and later extended by Glänzel and Czerwon (1996) who also applied the methodology. As applied to the *CMN* database, consider each composer *i* as a $n \times 1$ vector in the space of all *n* composers in the database. If a composer *k* among the *n* composers was an influence on *i*, then the *k*th component of the vector corresponding to composer *i* is set equal to 1, otherwise it is set equal to 0. Therefore, with respect to all composers in the database, each composer *i* is represented by a Boolean vector of 0’s and 1’s. The cosine similarity measure for a pair of composers (*i*, *j*), each represented by their own Boolean vectors *B*_*i*_ and *B*_*j*_, can then be computed as:

1 $$COS_{i,j} = \frac{{\sum\nolimits_{k = 1}^{n} {B_{k,i} \times B_{k,j} } }}{{\sqrt {\sum\nolimits_{k = 1}^{n} {\left( {B_{k,i} } \right)^{2} } } \sqrt {\sum\nolimits_{k = 1}^{n} {\left( {B_{k,j} } \right)^{2} } } }},$$

where subscript *k* in *B*_*k,i*_ indicates the *k*th component (of value 1 or 0) of vector *B*_*i*_. Thus, in essence, the cosine of the angle between the two vectors *B*_*i*_ and *B*_*j*_ gives a measure of association/similarity. The cosine similarity index ranges between 1 and 0, where 1 indicates that two composers are exactly identical and 0 indicates complete opposition. A value somewhere in the middle of the 0–1 range indicates degrees of independence of two composers. As discussed in Smith et al. (2015), when all the vectors are Boolean vectors, the null distribution of the cosine similarity under the assumption of independence between two composers is unknown and has a nonzero mean; in order to derive a statistical test for the cosine measure, a centralised cosine measure was proposed (Giller 2012). The centralised cosine measure is the cosine measure computed on the centralised vectors, with respect to the mean (average) vectors. Assuming that: $\overline{{B_{i} }} = {{(1} \mathord{\left/ {\vphantom {{(1} n}} \right. \kern-0pt} n})\sum\nolimits_{k = 1}^{n} {B_{k,i} }$ and $\overline{{B_{j} }} = {{(1} \mathord{\left/ {\vphantom {{(1} n}} \right. \kern-0pt} n})\sum\nolimits_{k = 1}^{n} {B_{k,j} }$, the centralised cosine measure is:

2 $$CSC_{i,j} = \frac{{\sum\nolimits_{k = 1}^{n} {\left( {B_{k,i} - \overline{{B_{i} }} } \right) \times \left( {B_{k,j} - \overline{{B_{j} }} } \right)} }}{{\sqrt {\sum\nolimits_{k = 1}^{n} {\left( {B_{k,i} - \overline{{B_{i} }} } \right)^{2} } } \sqrt {\sum\nolimits_{k = 1}^{n} {\left( {B_{k,j} - \overline{{B_{j} }} } \right)^{2} } } }}.$$

In order to establish a connection between this formula and the elements in Table 3, I now use a result in Smith et al. (2015) who proved that the centralised cosine measure can be computed as:

3 $$CSC_{i,j} = {{(ad - bc)} \mathord{\left/ {\vphantom {{(ad - bc)} {\sqrt[{}]{(a + b)(c + d)(a + c)(b + d)}}}} \right. \kern-0pt} {\sqrt[{}]{(a + b)(c + d)(a + c)(b + d)}}},$$

where *a*, *b*, *c*, *d* are the count of composers in the sets $CI_{i,j}$, $I_{i, - j}$, $I_{j, - i}$, and $C - CI_{i,j} - DI_{i,j}$ described above, and reported in Table 3.

It can be shown that values of the centralised cosine measure range from −1.0 to 1.0. A value of 1.0 indicates that two composers are identical. A value of −1.0 indicates that two composers are complete opposite. A value of 0 shows that two composers are independent (unassociated). A nonzero value of the centralised cosine measure might be due to randomness or actual association between composers. Unlike in the case of the ordinary cosine measure, there is a proper statistical significance test. Under the assumption that the size of the database *n* is large enough, the distribution of the centralised cosine measure (under the assumption of independence) is approximately normal, with mean 0 and variance 1/*n*. Therefore, the distribution of the centralised cosine measure can be converted into a standard normal distribution using the *Z*-score/statistics:

4 $$Z = {{CSC} \mathord{\left/ {\vphantom {{CSC} {\sqrt {1/n} }}} \right. \kern-0pt} {\sqrt {1/n} }} \Leftrightarrow Z = ABS(CSC\sqrt n ),$$

where *ABS* is the absolute value and *n* is the size of the database at hand, that is *n* = 500 for the personal musical influences database and *n* = 298 for the ecological categories database.^16^ Using the centralised cosine measure, Table 4 ranks composers in order of greater similarity to Debussy, on the basis of personal musical influences. The index identifies Ravel as the composer most similar to Debussy. The centralised cosine measure for Debussy and Ravel is 0.587. The corresponding *Z*-statistic is 13.119, which is greater than the critical value of 1.96 at a 5% significance level under the standard normal distribution. We can then reject the null hypothesis of no association between Debussy and Ravel.^17^

**Table 4:** Debussy versus other composers—similarity based on personal musical influences. *Source*: Computed by the author on the basis of data collected from ‘The Classical Music Navigator’ (Smith 2000)

CSC rank	Composers versus Debussy	CSC	*Z*-statistic	BID rank	Composers versus Debussy	BID
1	14. Debussy	1.000	22.361	1	14. Debussy	500.000
2	20. Ravel	0.587	13.119	2	20. Ravel	172.106
3	157. Enescu	0.439	9.815	3	157. Enescu	96.337
4	265. Koechlin	0.439	9.815	4	265. Koechlin	96.337
5	283. Indy	0.439	9.815	5	283. Indy	96.337
6	67. Villa-Lobos	0.433	9.686	6	67. Villa-Lobos	93.825
7	371. Moreno Torróba	0.389	8.708	7	371. Moreno Torróba	75.828
8	24. Rachmaninov	0.387	8.660	8	24. Rachmaninov	74.992
9	250. Glière	0.387	8.660	9	250. Glière	74.992
10	290. Duparc	0.370	8.284	10	290. Duparc	68.623
11	306. Lyadov	0.370	8.284	11	306. Lyadov	68.623
12	400. Gretchaninov	0.362	8.098	12	400. Gretchaninov	65.575
13	52. Franck	0.361	8.077	13	52. Franck	65.243
14	109. Granados	0.361	8.077	14	109. Granados	65.243
15	167. Chausson	0.361	8.077	15	167. Chausson	65.243
16	36. Sibelius	0.338	7.551	16	36. Sibelius	57.017
17	121. Glazunov	0.338	7.551	17	121. Glazunov	57.017
18	115. Bloch	0.335	7.482	18	115. Bloch	55.975
19	248. Mompou	0.335	7.482	19	248. Mompou	55.975
20	334. Jongen	0.335	7.482	20	334. Jongen	55.975
⋮	⋮			⋮	⋮	
181	480. Monk	0.090	2.017	181	480. Monk	4.068
⋮	⋮			⋮	⋮	
239	44. Hindemith	0.039	0.872	239	44. Hindemith	0.761
240	183. Bolcom	0.039	0.872	240	183. Bolcom	0.761
241	4. Schubert	0.034	0.767	241	110. Carter	0.712
242	126. Takemitsu	0.034	0.767	242	4. Schubert	0.588
243	191. Adams	0.034	0.767	243	126. Takemitsu	0.588
244	282. Rochberg	0.034	0.767	244	191. Adams	0.588
245	341. Kurtág	0.034	0.767	245	282. Rochberg	0.588
246	58. Sullivan	0.030	0.672	246	341. Kurtág	0.588
247	59. Cage	0.030	0.672	247	8. Handel	0.566
248	136. Henze	0.030	0.672	248	40. Purcell	0.566
249	10. Chopin	0.026	0.586	249	9. Haydn, J	0.518
⋮	⋮			⋮	⋮	
254	1. Bach, JS	0.002	0.034	254	202. Harrison	0.470
255	438. Oliveros	−0.010	0.215	255	287. Górecki	0.470
⋮	⋮			⋮	⋮	
269	459. Allegri	−0.010	0.215	269	349. Maderna	0.374
270	279. Boyce	−0.010	0.215	270	10. Chopin	0.344
⋮	⋮			⋮	⋮	
463	349. Maderna	−0.027	0.612	463	407. La Rue	0.046
464	131. Hummel	−0.027	0.612	464	418. Clérambault	0.046
465	293. Nono	−0.027	0.612	465	419. Couperin, L	0.046
466	29. Berlioz	−0.027	0.612	466	421. Gombert	0.046
467	311. Nyman	−0.029	0.649	467	432. Anderson	0.046
⋮	⋮			⋮	⋮	
471	287. Górecki	−0.031	0.685	471	450. Piccinni	0.046
472	50. Bernstein	−0.031	0.685	472	459. Allegri	0.046
473	178. Davies	−0.031	0.685	473	466. Moore	0.046
474	202. Harrison	−0.031	0.685	474	467. Stamitz, J	0.046
475	9. Haydn, J	−0.032	0.719	475	479. Martini	0.046
476	186. Krenek	−0.032	0.719	476	487. Sheppard	0.046
477	124. Boulez	−0.032	0.719	477	492. Clemens	0.046
478	8. Handel	−0.034	0.752	478	495. Bruhns	0.046
479	40. Purcell	−0.034	0.752	479	499. Lauro	0.046
480	110. Carter	−0.038	0.844	480	1. Bach, JS	0.001

The number in front of a composer’s name gives his ranking (in terms of importance), as defined in the *CMN*. This is the primary ranking discussed in next section

As said above, when *CSC* takes a value of 0, this means that the two composers under consideration are ‘independent’ (unassociated). So, a negative value for *CSC* suggests that the composers are negatively associated. But what is the exact meaning of this? Recall that the centralised cosine measure is based on Boolean vectors. The Boolean vector for Debussy, *B*_*i*_ = *B*_Debussy_, is a (500 × 1) vector of components *B*_*k,*Debussy_ each equal to ‘1’ or ‘0’ depending on whether a composer $k \in C$ had or not a reported musical influence on Debussy. The Boolean vector for Carter follows an analogous definition. If the sets of personal musical influences on Debussy and Carter are such that *B*_*k,*Carter_ is more often 1 (or 0) when *B*_*k,*Debussy_ is 0 (or 1), then *CSC* will take a negative value and this suggests that Carter may have (deliberately or not) rejected composers that had a musical influence on Debussy while being influenced by others that had no reported musical influence on Debussy. This property of the centralised cosine measure provides a more sensitive measure of ‘similarity’ than the binomial index described in the “Appendix” (and previously used by Smith and Georges 2014, 2015) as it also tracks composers who (consciously or not) attempted to ‘differentiate’ themselves from others.^18^

**Fig. 4:**
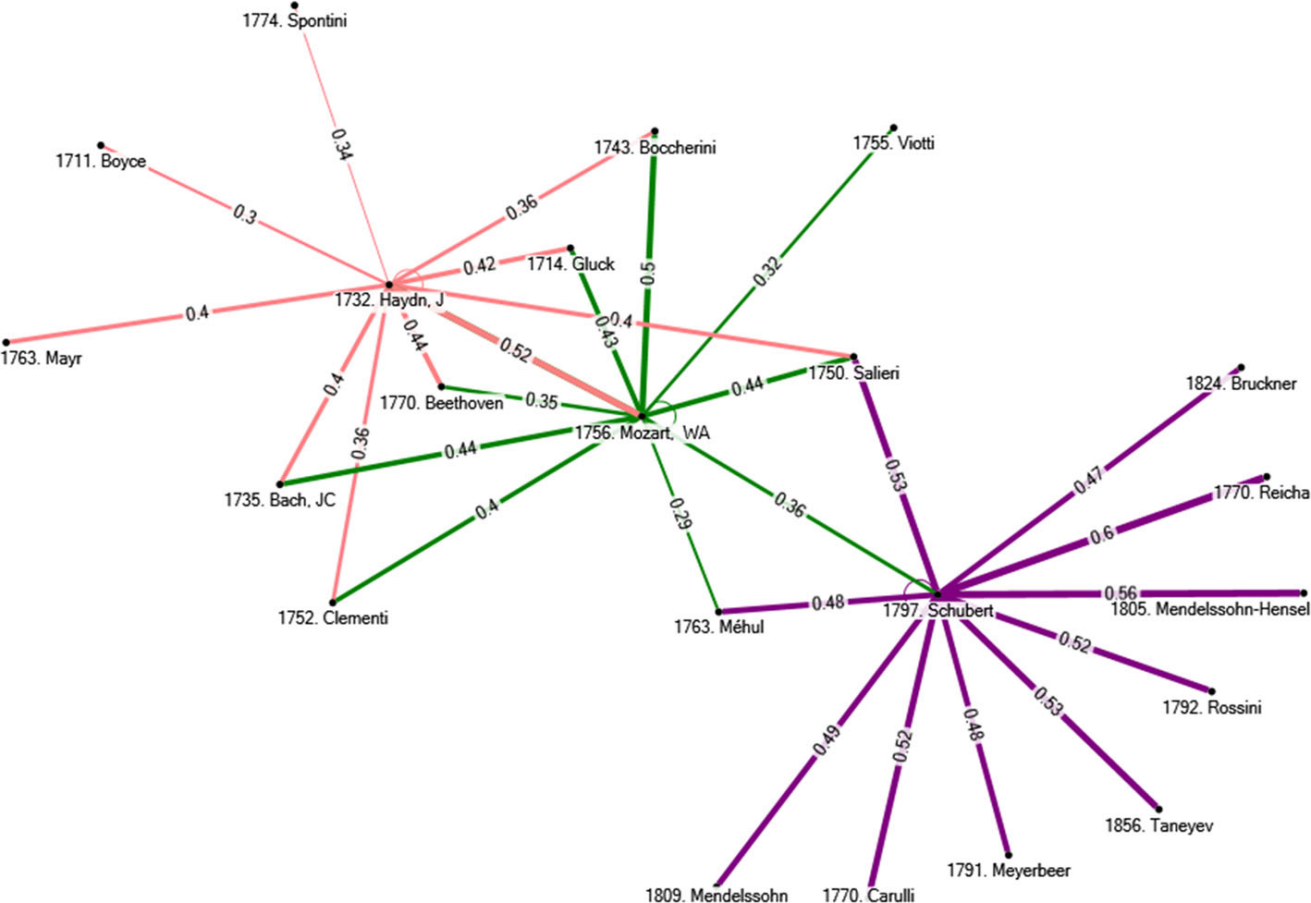
Ten most similar composers to J. Haydn, W. A. Mozart, and Schubert on the basis of personal musical influences. *Notes* (1) The *number* in front of a composer’s name in figure corresponds to his date of birth. (2) The *number* on the edge linking any pair of composers gives the centralised cosine similarity index (on the basis of personal musical influences) between the two composers. Note that the width of the edge also proxies the degree of similarity

**Fig. 5:**
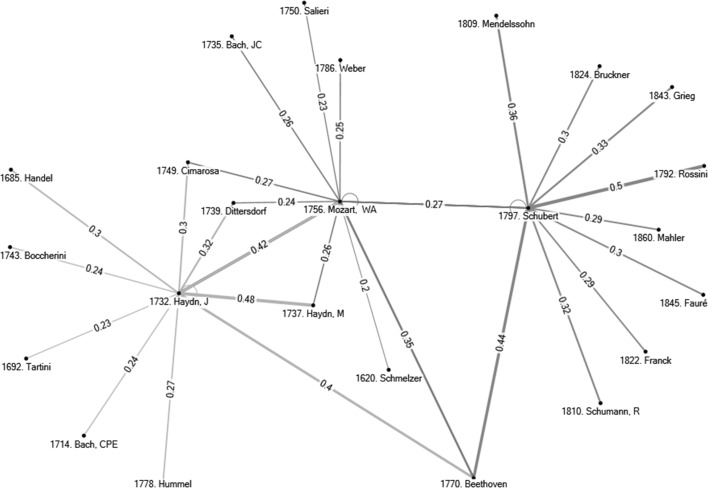
Ten most similar composers to J. Haydn, W. A. Mozart, and Schubert on the basis of ecological characteristics. *Notes* (1) The number in front of a composer’s name in figure corresponds to his date of birth. (2) The number on the edge linking any pair of composers gives the centralised cosine similarity index (on the basis of ecological characteristics) between the two composers. Note that the width of the edge also proxies the degree of similarity

For all 500 subject composers, two tables of similarity indices have been generated, one on the basis of the personal musical influences database (as done in the example for Debussy), and one that is based on the 298 ecological characteristics database. The large number of indices computed ($2 \times 500 \times 500$) forces us to report average results for subsets of composers and specific results for a few composers only. Before doing this in next section, observe Figs. 4 and 5. Figure 4 gives the ten most similar composers to J. Haydn, Mozart, and Schubert, on the basis of personal musical influences using the centralised cosine similarity measure developed in this section. Observe the differences between Figs. 2 and 4. Figure 2 provides composers who had a reported influence on these three subject composers. The assumption in the first section was that the larger number of common personal influences between W. A. Mozart and J. Haydn would cause (or even explain) the higher similarity of styles between these two composers than between Mozart and Schubert, let alone J. Haydn and Schubert. Figure 4 confirms that J. Haydn and Mozart have a higher centralised cosine similarity index (0.52) than Mozart and Schubert (0.36) or Haydn and Schubert (0.26).^19^ Figure 5 gives the 10 most similar composers to J. Haydn, W. A. Mozart, and Schubert on the basis of ecological characteristics. Two things are worth noticing. First, when comparing similarities on the basis of personal musical influences and ecological data there are only three common names in the two lists of the 10 most similar composers to J. Haydn (i.e., Mozart, Beethoven, Boccherini), three common names in the lists for Schubert (i.e., Rossini, Mendelssohn, Bruckner), and five common names in the two lists related to Mozart (J. Haydn, JC Bach, Salieri, Schubert, Beethoven). This is not surprising because personal musical influences and ecological data provide two different perspectives on the concept of similarity. Second, observe that most composers similar to Mozart and to Haydn are, in both lists, Classical period composers. However, many composers similar to Schubert on the basis of ecological characteristics are Romantic period composers (R. Schumann, C. Franck, Grieg, Fauré, Mahler—all composers born quite after Schubert). Yet, the similarity list based on personal musical influences (lineage) suggests that Schubert is strongly associated to older composers of the Classical period (e.g., Reicha, Salieri, Carulli, Méhul, and Rossini). This confirms the insight of the previous section—Exploiting the conflicting results generated by the two databases is a useful approach to detect transitional-period composers such as Schubert, whose lineage is still anchored in the Classical period while his musical ecological niche pulls him towards the Romantic period.^20^ This explains to some extent music ‘speciation’ and evolution—a large number of Schubert’s compositions ‘sound’ different from the music of Mozart and Haydn, even if Schubert’s influences network (lineage) remains anchored in the Classical period. This also suggests that a presentation analogous to Table 2 could help us detect music speciation and evolution. This is explored further in the following section.

## Selected statistical results and discussion

**Fig. 6:**
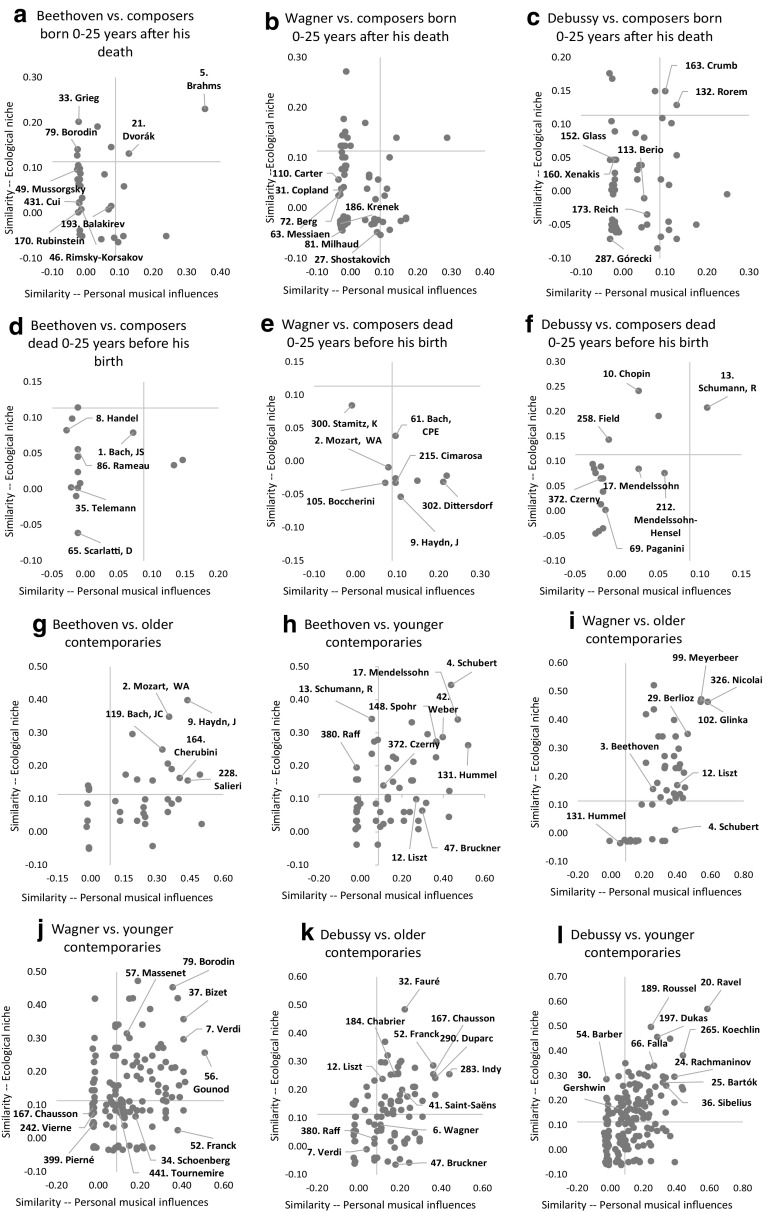

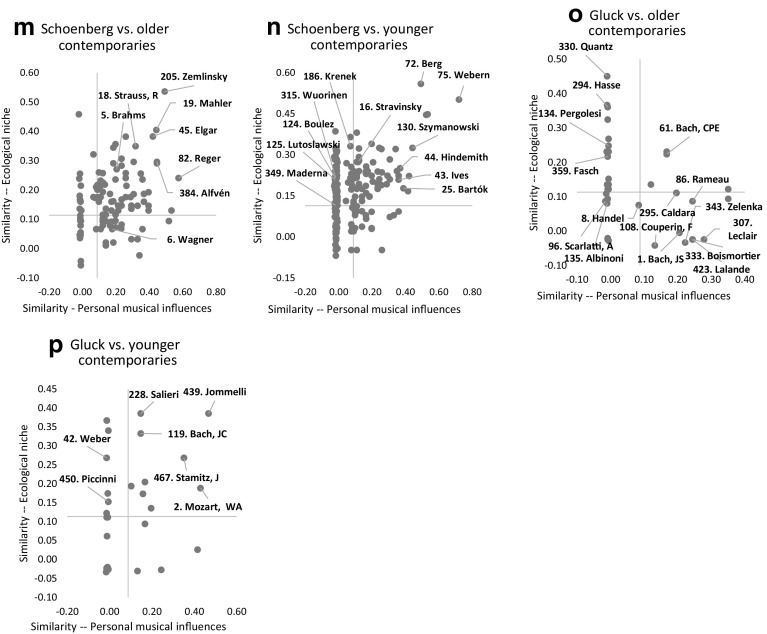
A few selected ‘subject’ composers. *Notes* (1) Each dot in these figures is a vector that represents an ‘object’ composer, located relative to the ‘subject’ composer of the figure, according to the values of two similarity indices based on: (1) personal musical influences (lineage) on the *X*-axis and (2) musical ecological niches on the *Y*-axis. The axes do not cross at the origin but at the critical values delimiting statistically-significant similarity index values (above) versus independence/dissimilarity (below). (2) The number in front of a composer’s name is a ranking which reflects the importance of this particular composer. This is the primary ranking established in ‘The Classical Music Navigator’ (Smith 2000), and also discussed in main text of this section

Built from the perspective of a ‘subject’ composer, Fig. 6a–p plot vectors (dots) representing other composers located relative to the ‘subject’ composer according to their similarity in terms of personal musical influences (*X*-axis) and ecological characteristics (*Y*-axis). For purpose of clarification, we will refer to these ‘other’ composers—the dots in Fig. 6a–p—as ‘object’ composers in the sense that they are compared to one unique ‘subject’ composer. For example, in Fig. 6a Beethoven is the ‘subject’ of the analysis and Brahms, Dvořák, etc. are ‘object’ composers located (with dots) relative to Beethoven. Furthermore, ‘object’ composers are grouped into four categories according to an age relationship with the ‘subject’ composer: 1. Composers dead 0–25 years before the birth of the ‘subject’ composer, 2. Older contemporary composers, 3. Younger contemporary composers, and 4. Composers born 0–25 years after the death of the ‘subject’ composer. See Fig. 6a, d, g, h, respectively, for ‘subject’ composer Beethoven.

Note that the two axes in all panels of Fig. 6 have been drawn at their critical significant values at 5%. Given Eq. 4, the *Z*-statistic is at its critical value when *Z* = $ABS\left( {CSC \times \sqrt n } \right)$ = 1.96. The value for *n* is 500 in the case of the influences network database, and 298 in the ecological characteristic database. Thus, the critical values are $CSC_{\text{c}} = \pm 1.96/\sqrt {500} = \pm 0.0877$ and $CSC_{\text{c}} = \pm 1.96/\sqrt {298} = \pm 0. 1 1 3 5$, respectively. The four quadrants delimited by the two positive critical values correspond to the four cells in Table 2. Thus, the word ‘high’ in Table 2 is now assumed to represent a statistically significant positive association between ‘object’ and ‘subject’ composers, and the word ‘low’, no statistically significant association.^21^ In some panels of Fig. 6, we can also see vertical and horizontal spikes of dots at the origin (zero). These dots represent independence (along one of the two criteria). Observe therefore four cases: (1) ‘Object’ composers who score high on both indices are located in the North-East quadrant and are considered to be very similar to the ‘subject’ composer. (2) ‘Object’ composers who score low on both indices are located in the South-West quadrant. Their association to the ‘subject’ composer is statistically insignificant on both criteria and they are considered to be most dissimilar to the ‘subject’ composer. (3) ‘Object’ composers who score high on the personal influence index, but low on the ecological index, with respect to the ‘subject’ composer, are located on the South-East quadrant. Their ecological niches are different from the one of the ‘subject’ composer, even if they share a common lineage of personal musical influences. As we argued before, this may be a sign of music speciation and evolution. (4) ‘Object’ composers who score low on the personal influence index but high on the ecological index with respect to the ‘subject’ composer are located in the North-West quadrant. Despite no or little common personal lineage with the ‘subject’ composer, they have developed a somewhat similar sound by composing in musical niches that share many ecological characteristics. Using evolutionary biology terminology, this could be a sign of ‘convergent evolution’.

Of course, a high positive value for a similarity index reveals a significant association between a pair of composers, but does not imply causality. Still, by grouping composers on the basis of an age relationship with the ‘subject’ composer we can somehow identify the antecedent or ‘causality in similarity’. For example, if an ‘object’ composer was located in the South-East quadrant but died before the birth of the ‘subject’ composer, then music speciation/evolution should be attributed to the ‘subject’ composer. The latter distanced himself from the former by composing in a different musical/ecological niche. However, under the same South-East location, music evolution/speciation should be attributed to the ‘object’ composer if he was born after the death of the ‘subject’ composer. Extending this reasoning in the case of contemporary composers (both alive at one point in time) is of course ambiguous. A much younger contemporary composer is likely to be the one imitating or differentiating oneself from the older composer. But some degree of cross-imitation must be expected from composers of similar ages.

Figure 6a–p apply this graphical approach to a few composers such as Gluck, Beethoven, Wagner, Debussy, and Schoenberg, and I discuss their specifics later on in this section. As one cannot make general statements about Western classical music evolution, a gigantic undertaking of a major art form, based on an analysis of just five ‘subject’ composers, I first start by establishing some general observations. Tables 5a–c present statistics covering individual, some subsets, and all of the 500 composers included in the database. Table 5a is essentially equivalent to Fig. 6. For example, Table 5a is divided into four panels corresponding to the four age relationships between ‘subject’ and ‘object’ composers. Table 5a also gives the density in each quadrant (North-East, South-East, North-West and South-West), that is, it computes with respect to a ‘subject’ composer, frequencies of occurrence of ‘object’ composers located in each quadrant. Table 5a reports results for five specific ‘subject’ composers (Monteverdi, Gluck, Beethoven, Debussy, and Schoenberg).^22^ However, this computation was done for *all* 500 ‘subject’ composers and Table 5b reports *average* results over all 500 ‘subject’ composers. The first column in the first panel of Table 5b (‘subject’ composer vs. composers dead 0–25 years before the birth of the ‘subject’ composer) gives the mean (and standard deviation in brackets) of these frequencies computed over all 500 ‘subject’ composers—4, 11, 25 and 60% for, respectively, the North-East, South-East, North-West, and South-West quadrants. The results illustrate that, *on average*, composers strongly differentiate from recently dead composers. Sixty percent of them compose in a different ecological niche (from the one associated to dead composers) and have no significant similarity on the basis of personal musical influences (South-West quadrant). Only 4% of them are statistically similar to those dead composers with respect to ecological niche and personal influences (North-East quadrant).^23^ Finally, observe the much higher density in the North-East quadrants and lower density in the South-West quadrants in the first column of panels 2 and 3, where ‘subject’ composers are compared to either older or younger contemporaries, respectively. This suggests an overall larger tendency for cross-imitation between pairs of contemporaries (higher similarity in personal musical influences and ecological niches).

**Table 5:** Some statistical results

(a) Statistical results for ‘innovative’ or style-changing composers										
	1. Subject composer versus composers dead 0–25 years before the birth of the subject composer					2. Subject composer versus older contemporaries				
	53. Monteverdi	68. Gluck	3. Beethoven	14. Debussy	34. Schoenberg	53. Monteverdi	68. Gluck	3. Beethoven	14. Debussy	34. Schoenberg
NorthEast	0.10	0.10	0.00	0.05	0.30	0.53	0.15	0.36	0.44	0.54
SouthEast	0.00	0.20	0.13	0.00	0.50	0.11	0.26	0.42	0.26	0.20
NorthWest	0.60	0.20	0.07	0.16	0.05	0.21	0.36	0.08	0.09	0.13
SouthWest	0.30	0.50	0.80	0.79	0.15	0.16	0.23	0.14	0.21	0.13

	3. Subject composer versus younger contemporaries					4. Subject composer versus composers born 0–25 years after the death of the subject composer				
	53. Monteverdi	68. Gluck	3. Beethoven	14. Debussy	34. Schoen-berg	53. Monteverdi	68. Gluck	3. Beethoven	14. Debussy	34. Schoenberg
NorthEast	0.33	0.23	0.33	0.35	0.28	0.13	0.04	0.06	0.03	0.00
SouthEast	0.00	0.11	0.28	0.19	0.10	0.00	0.13	0.12	0.19	0.50
NorthWest	0.39	0.17	0.13	0.13	0.42	0.87	0.17	0.15	0.05	0.00
SouthWest	0.28	0.49	0.26	0.32	0.20	0.00	0.65	0.67	0.72	0.50

(b) Statistical results for ‘top’ subject composers compared to all 500 composers												
	1. Subject composer versus composers dead 0–25 years before the birth of the subject composer						2. Subject composer versus older contemporaries					
	ALL	TOP 20S	TOP 20M	TOP 20iS	TOP 50S	TOP 100S	ALL	TOP 20S	TOP 20M	TOP 20iS	TOP 50S	TOP 100S
NorthEast	0.04 (0.08)	0.10	0.08	0.08	0.09	0.08	0.28 (0.19)	0.46	0.43	0.47	0.44	0.41
SouthEast	0.11 (0.17)	0.32	0.26	0.30	0.26	0.21	0.19 (0.16)	0.31	0.31	0.30	0.27	0.25
NorthWest	0.25 (0.29)	0.12	0.14	0.15	0.14	0.20	0.25 (0.20)	0.08	0.10	0.09	0.12	0.16
SouthWest	0.60 (0.33)	0.46	0.52	0.48	0.52	0.52	0.28 (0.19)	0.15	0.16	0.14	0.17	0.18

	3. Subject composer versus younger contemporaries						4. Subject composer versus composers born 0–25 years after the death of the subject composer					
	ALL	TOP 20S	TOP 20M	TOP 20iS	TOP 50S	TOP 100S	ALL	TOP 20S	TOP 20M	TOP 20iS	TOP 50S	TOP 100S
NorthEast	0.25 (0.16)	0.32	0.33	0.32	0.31	0.30	0.04 (0.08)	0.07	0.06	0.07	0.07	0.06
SouthEast	0.20 (0.20)	0.27	0.22	0.23	0.24	0.23	0.11 (0.15)	0.16	0.15	0.17	0.14	0.14
NorthWest	0.26 (0.20)	0.14	0.19	0.21	0.18	0.20	0.24 (0.33)	0.13	0.18	0.14	0.19	0.20
SouthWest	0.29 (0.19)	0.27	0.26	0.23	0.27	0.27	0.61 (0.55)	0.64	0.60	0.62	0.60	0.60

(c) Statistical results for composers of specific music periods compared to all 500 composers												
	1. Subject composer versus composers dead 0–25 years before the birth of the subject composer						2. Subject composer versus older contemporaries					
	ALL	Renaissance	Baroque	Classical	Romantic	Modern	ALL	Renaissance	Baroque	Classical	Romantic	Modern
NorthEast	0.04	0.00	0.07	0.02	0.06	0.03	0.28	0.44	0.31	0.23	0.35	0.21
SouthEast	0.11	0.00	0.02	0.05	0.16	0.14	0.19	0.02	0.06	0.16	0.25	0.23
NorthWest	0.25	0.89	0.59	0.29	0.18	0.09	0.25	0.49	0.45	0.27	0.17	0.19
SouthWest	0.60	0.11	0.33	0.64	0.59	0.75	0.28	0.05	0.18	0.34	0.23	0.37

	3. Subject composer versus younger contemporaries						4. Subject composer versus composers born 0–25 years after the death of the subject composer					
	ALL	Renaissance	Baroque	Classical	Romantic	Modern	ALL	Renaissance	Baroque	Classical	Romantic	Modern
NorthEast	0.25	0.30	0.21	0.22	0.22	0.28	0.04	0.04	0.03	0.04	0.04	0.07
SouthEast	0.20	0.04	0.08	0.22	0.23	0.23	0.11	0.01	0.04	0.15	0.14	0.15
NorthWest	0.26	0.54	0.41	0.23	0.17	0.24	0.24	0.59	0.36	0.16	0.12	0.25
SouthWest	0.29	0.13	0.30	0.33	0.38	0.25	0.61	0.37	0.58	0.65	0.71	0.52

Columns ‘Top 20S’, ‘Top 50S’ and ‘Top 100S’ stand for Smith (2000) primary rankings based on his Top 100 list. ‘Top 20M’ stands for Murray (2003) ranking of the Top 20 composers. Finally, ‘Top 20iS is Smith (2000) secondary ranking of the Top 20 most ‘influential’ composers

We pursue the analysis by considering subsets of ‘subject’ composers regrouped into rankings such as Top-20 (or Top-50, or Top-100) most ‘important’ composers.^24^ We also grouped them by periods such as all 48 Renaissance composers included in the Classical Music Navigator (*CMN*) database, all 50 Baroque composers, all 57 Classical, all 146 Romantic, and all 195 Modern composers.^25^ Of the three rankings used here, the first one is the ‘primary’ ranking of the Top-100 composers computed by Smith (2000) in the *CMN*, from which Top-20 and Top-50 rankings are also derived (and referenced in Table 5b as TOP 20S, TOP 50S and TOP 100S).^26^ The second one is Smith’s ‘secondary’ ranking of most ‘influential’ composers, based on the list and the (primary) ranking of those composers who were influenced by the composer under study (TOP 20iS in Table 5b).^27^ The third one (TOP 20M in Table 5b) is the Top-20 ranking proposed by Murray (2003).^28^ In the following, I only discuss results for Top-20 composers according to the primary ranking of Smith (TOP 20S), because other rankings give roughly similar results. Hence results are robust and do not depend on the method underlying the construction of these rankings. Compare first and second columns in panel 2 of Table 5b (Columns ALL and TOP 20S) and think of the mean across all 500 ‘subject’ composers (first column) as the result pertaining to an ‘average’ subject composer. We therefore see that Top-20 ‘subject’ composers have (on average) denser North-East and South-East quadrants than the average ‘subject’ composer (0.46 > 0.28 and 0.31 > 0.19). This suggests that the creative process of Top-20 composers (even more so than for an average composer), is not due to genius alone but is based on personal musical influences, in particular a strong similar lineage (or network of personal influences) with older contemporaries. This reminds the much-quoted expression attributed to Isaac Newton: “if I have seen further, it is by standing on the shoulders of giants.” Concentrating more specifically on the South-East quadrant, we observe that it is denser for Top-20 ‘subject’ composers than for the average ‘subject’ composer (31 vs. 19%). According to our typology in Table 2, this suggests that major composers, while also sharing personal musical influences with older contemporaries, contributed more than the ‘average’ composer to music evolution by composing in a different (i.e., new) musical ecological niche, which, in turn, made them sound ‘different’ from the average composer. On the other hand, the North-West quadrant for the average ‘subject’ composer is denser than the one corresponding to Top-20 ‘subject’ composers (25 vs. 8%). This means, first, that the ‘average’ composer has a distinct personal lineage (from the one of older contemporary composers), suggesting that the ‘average’ composer is somewhat isolated, or perhaps less well-connected (than Top-20 composers) to the network of key influences. Secondly, this means that the ‘average’ composer is more likely to share the musical ecological niche of older contemporaries, eventually producing music that sounds somewhat similar (convergent evolution), and as such contributing less to the evolution of Western classical music.

Although panels 1 and 3 of Table 5b can generally be interpreted along similar lines as panel 2, panel 4 brings an interesting twist. Imitation or differentiation, in panel 4, must be attributed to the ‘object’ composer as the ‘subject’ composer is dead. Panel 4 therefore means that ‘object’ composers are more likely to differentiate themselves (or at least be independent) from Top-20 composers than from an ‘average’ composer (64 vs. 61%).^29^ This perhaps reflects the idea that new generations try to differentiate themselves in particular from top (dead) composers, for fear of being categorised as ‘epigones’ by music historians and eventually forgotten by the public.^30^

One problem with our focus on Top-20 ‘subject’ composers is that they are not necessarily ‘innovators’ or ‘transitional’ composers, (i.e., composers located on the diagonal in Fig. 1 as identified by musicologists). For example, few musicologists would consider J.S. Bach or W. A. Mozart, two major composers, to be genuine innovators. An alternative strategy is therefore to compare innovators and/or transitional figures with composers of the music period from which they progressively diverged, for example, by comparing Monteverdi to all Renaissance composers, Gluck versus all Baroque composers, Beethoven versus all Classical composers, Debussy and Schoenberg versus all Romantic composers. We therefore propose to compare statistical results for specific ‘innovators’ in Table 5a with results for the ‘average’ subject composer of a specific period in Table 5c. Focusing on panel 2 in both tables, we see that the South-East quadrant for specific ‘innovators’ in Table 5a is denser than the quadrant corresponding to the ‘average’ composer of the period from which they progressively diverged. In the case of Beethoven, 42% of his older contemporaries fall in the South-East quadrant while the corresponding number is just 16% for the ‘average’ classical composer. This not only means that Beethoven was better connected (than the ‘average’ classical composer) to older contemporaries in terms of personal musical influences (i.e., ‘standing on the shoulders of giants’), but also that he was progressively composing in a different musical ecological niche (than the one of the ‘average’ classical composer), leading to a change of sound in classical music and opening the way to the Romantic period. This is also true for Monteverdi (11%) versus the ‘average’ Renaissance composer (2%) or Gluck (26%) versus the ‘average’ Baroque composer (6%). This, however, is just marginally true for Debussy (vs. the ‘average’ Romantic composer), and not true for Schoenberg (20 vs. 25% for the ‘average’ Romantic composer).^31^ One difficulty is, of course, the concept of an ‘average’ Romantic composer who would be representative of a rather long period divided itself in very distinct sub-periods—early, middle and late Romantic periods—each having their own ‘innovators’ or transitional composers. Besides, it is also informative to recall that Schoenberg felt that his early music would prove his understanding of and respect for tradition.^32^ This perhaps explains our results in panel 2 of Table 5a (or in Fig. 6m) that characterize Schoenberg as building on the romantic tradition (very dense North-East quadrant −54%) instead of being exclusively characterised as an innovator.

After these general observations, I now pursue with a few specific results related to Fig. 6a–p for ‘subject’ composers Gluck, Beethoven, Wagner, Debussy, and Schoenberg (of which Gluck, Beethoven, Debussy and Schoenberg are viewed by musicologists as innovators and ‘transitional’ composers, and therefore positioned on the diagonal in Fig. 1). The objective is to demonstrate that our results, based on a statistical methodology, confirm many facts well-known to musicologists.

First, observe again that after the death of the ‘subject’ composer, there is a strong tendency for newer generations of composers to seek different personal lineages and/or musical ecological niches (i.e., the North-East quadrants of Fig. 6a–c, have a very low density of dots relative to other quadrants, in particular the South-West quadrant). Figure 6c shows that twentieth century composers Xenakis, Berio, Reich and Glass who were born 0–25 years after the death of Debussy, are quite different from him on both criteria. See also Fig. 6a for Beethoven and Fig. 6b for Wagner. Although this confirms the general result observed previously, it is worth emphasizing that this is a differentiation away from ‘subject’ composers (such as Beethoven, Wagner, or Debussy) who are known to have had direct influences on younger contemporary composers. Hence, a strong process of music evolution and differentiation operates over time, across new generations. Of course, there are exceptions. A composer such as Brahms, born after the death of Beethoven, appears in the North-East quadrant of Fig. 6a, suggesting strong similarities with Beethoven. And, as is well known, Brahms’ First Symphony (from 1876) has often been compared to the Ninth Symphony of Beethoven (1824).^33^ Music evolution and differentiation can also be viewed from another side, when observing graphs of ‘object’ composers who were dead before the birth of the ‘subject’ composer. We observe in Fig. 6d–f a low density of ‘object’ composers in the North-East quadrant, which suggests that the ‘subject’ composer (respectively, Beethoven, Wagner, and Debussy) distanced himself from past generations of composers in terms of musical ecological niche and/or personal lineage.

Second, results are quite different from those reported above when considering ‘contemporary’ composers (Fig. 6g–p). In this case, we typically observe a large density of dots in the North-East quadrants, suggesting a process of imitation. For example, we see the common personal lineage and ecological niches of Beethoven with older contemporaries such as J. Haydn and W. A. Mozart (Fig. 6g). Then, it is the turn of younger contemporaries such as Hummel, Schubert, Mendelssohn, to also ‘imitate’ Beethoven to some extent (Fig. 6h). We see the extent to which Wagner’s music is both a product of his time and a music that has been imitated, with a large density in the North-East quadrant for older composers (Berlioz, Meyerbeer, Glinka, Nicolai in Fig. 6i) and younger contemporaries (Gounod, Borodin, Bizet, Massenet in Fig. 6j). We see a strong similarity of Debussy with some of his older contemporaries in Fig. 6k (Franck, Fauré, Chabrier, Chausson), and we see Ravel and Roussel subsequently embracing Debussy’s impressionism (Fig. 6l). We see the middle and late Romantic heritage of Schoenberg (e.g., Brahms, R. Strauss, Mahler, Reger) in Fig. 6m and then we see Berg and Webern developing the innovative dodecaphonic (or twelve-tone) method of composition of Schoenberg (Fig. 6n). We finally see in the North-East quadrant of Fig. 6p that Gluck and Jommelli (both born in 1714) are very similar. Gluck’s reforms of the opera will be discussed shortly. However, note that Jommelli is also known for his reforms of the Italian opera, so much so that he has been called the ‘Italian Gluck’ (Grout and Williams 2002).

Third, relationships among contemporaries are not just limited to a process of imitation; we also see a process of differentiation and evolution among them as the South-West and South-East quadrants are also densely populated in Fig. 6g–p). According to our typology, Gluck’s music is different from earlier Baroque contemporaries such as A. Scarlatti and, later on, Handel (South-West quadrant of Fig. 6o). Indeed, Gluck’s reforms of the opera of the mid-eighteenth century was a reaction to the excesses of ‘pre-reforms’ Baroque *opera seria* (and the virtuosic display of *da capo* aria) of composers such as A. Scarlatti and followers.^34^ He abolished vocal virtuosic excess for its own sake so that the music would serve the needs of the drama, that linguistic elements took place over purely musical considerations, that realism was privileged over fantasy or irrationality. Gluck’s operas, despite all his reforms, also follow the conventions of the older French *Tragédie Lyrique*, including the use of librettos in French language, which tends to explain the common lineage with Rameau and other French baroque composers located in the South-East part of Fig. 6o, despite the obvious evolution from their music.^35^

Continuing with other composers who changed the sound of music, we see that Liszt, a younger contemporary of Beethoven, has developed a music different from the one of Beethoven despite having a similar lineage (Fig. 6h, South-East quadrant).^36^ Much of the symphonic writing (‘traditional’, ‘non-programmatic’, ‘multi-movement’ symphony) fell out of fashion after Beethoven’s Ninth symphony (1824). From that point onwards, the last symphony of Schubert, and those of Mendelssohn and Schumann, however magnificent they are, could only be regarded as the works of epigones. And symphonies composed yet later on, in the 1850s and 1860s, by conservative composers such as Anton Rubinstein, Carl Reinecke, Max Bruch, or Joachim Raff have not successfully survived the repertoire. But, Liszt symphonic poems, by combining the ‘poetic’ and the ‘musical’, were what had to devolve if progress was to be gained.^37^ We also see music evolution in the case of Bruckner (Fig. 6h, South-East quadrant).^38^

Music speciation/evolution is also observed in the case of Franck and Schoenberg as younger contemporaries of Wagner (Fig. 6j, South-East quadrant). The influence of Wagner in the second half of the nineteenth century, although enormous, was selectively transformed. In France, the Belgium-born César Franck was the founding figure of a self-consciously French school of composition (Henri Duparc, Vincent d’Indy, Ernest Chausson, Gabriel Pierné, Guy Robartz, Charles Tournemire, Louis Vierne, Guillaume Lekeu, etc. See some of these names in Fig. 6j) that was distinctly French and progressively independent of German influence. In their symphonic works, these musicians challenged the longstanding Austro-German dominance of serious instrumental genres and cultivated a distinctly French musical voice (Seto 2012). The fact that Chausson is located in the South-West quadrant of Fig. 6j is revealing, as he wrote on several occasions (see Seto 2012) on his need to ‘dewagnerize’—a clear desire to differentiate himself from Wagner.^39^ As a further example, consider Schoenberg. As we saw earlier, he positioned himself as an heir of middle- and late-German romantic composers. See Fig. 6m and the references to Brahms, Mahler and R. Strauss. But around 1910 Schoenberg made his final break with tonality and differentiated himself through the development of dodecaphony (twelve-tone music/serialism) in the 1920s, one of the most polemic characteristics of twentieth-century music. This may explain Schoenberg’s position (relative to Wagner) in the South-East quadrant of Fig. 6j (or the mirrored result in Fig. 6m).

Finally, recall that the North-West quadrant was referred to as ‘convergent evolution’ (see also Table 2) in analogy to evolutionary biology. In our social and music context, this should not be interpreted too literally. That an ‘object’ composer is located in this quadrant simply means that he is sharing the ecological niche but not the lineage (personal musical influences) of the ‘subject’ composer. It could be that the ‘object’ composer is peripheral, that is, somewhat isolated in the network of personal musical influences. Or that, perhaps for geographical, social, or even partisan reasons, the networks of influences of the pair of composers do not overlap. Yet, this pair of composers may share the same musical niche because, in the case of Western classical music where the time frame is short and the spatial frame is small, any composer is aware of (and may compose in) the musical niche in which any other is composing. We see perhaps ‘convergent evolution’ in Fig. 6k (North-West quadrant) for U.S.-born Samuel Barber, a (much) younger contemporary of Debussy who, despite some American feel of his music, was rather isolated over there, and composed in an ecological niche (concertos, symphonies, opera) that was much closer to the late-Romantic European composers than the ecological niche (including jazzy elements and film music) of U.S. composers of his time such as A. Copland or L. Bernstein. We also see Gershwin whose composition, *An American in Paris*, reflects the journey that he had consciously taken as a composer. As cited in Hyland (2003), Gershwin declared with respect to this composition: “The opening part will be developed in typical French style, in the manner of Debussy and *Les Six*, though the tunes are original”.^40^ And despite all the jazzy elements of his music, his piano Concerto in *F* was criticised for being too much related to the work of Debussy. Despite Gluck’s opera reforms mentioned earlier, and his separate network of influences (including his partisans opposed to the famous poet and librettist Metastasio and his circle of *opera seria* composers using dazzling artifices), he was part of the transition between Baroque and Classical Periods, sharing the ecological niche of many composers (e.g., Fasch, Hasse and Pergolesi in Fig. 6o and Piccinni in Fig. 6p) who were also contributing to mid-eighteenth century stylistic changes, suggesting a convergent evolution.^41^

## Conclusion and future research

This paper uses two databases, the personal influences and the ecological categories databases extracted from the *CMN,* to test, statistically, for similarity between pairs of composers, using the centralised cosine similarity index. Each of these two databases permits to capture one aspect of similarity across pairs of composers. As such, this is a contribution to the music information retrieval research. However, this paper goes one step further by using the two similarity rankings conjointly in order to generate a typology of cases that permits to explore music imitation and differentiation, music ‘speciation’ and ‘convergent evolution’. That results in the fourth section corroborate many facts well known to musicologists is indicative of a sound database and methodology. This said, although there is scope for a true evolutionary model of Western classical music, including the construction of a phylogenetic tree, there are also challenges. In biological systematics, one is typically given some group of species (from within a large genus), and data on some number of their adaptive traits (plus external knowledge on which traits are viewed as more primitive to the others). Then, various algorithms have been developed to produce a family tree having the most likely chance of accurately reflecting speciation patterns over time. But in that instance, there is the useful simplification that each species comes from only one other, whereas in the Western classical music context, the ‘events’ (particular composers) are the product of multi-influence.

Hence, at this stage, it is best to see our work as preliminary background. First, it will take some time to sort through the numerous results obtained with the methodology introduced in this paper. Second, there is a need to improve this framework using a finer analysis, one that would introduce specific sub-periods (early, middle, and late Romantic periods, subdivisions of the twentieth century, etc.), and that would consider additional age categories among contemporaries (not just older vs. younger contemporary composers). Third, current results and their limitation are also driven by the information available in the *CMN*. One limitation is that the *CMN* data suffer from some spottiness, as many of the less significant composers on the list of 500 remain incompletely studied or commented upon. Musicological research on composers is an ongoing effort and newly discovered influences from (and on) lesser composers must progressively be included in the *CMN*. A large-scale literature review should reduce this problem and would permit to improve our narrative of Western classical music evolution based on statistical analysis and methods developed in biosystematics, scientometrics and bibliometrics.

## Figures and Tables

**Fig. 7:**
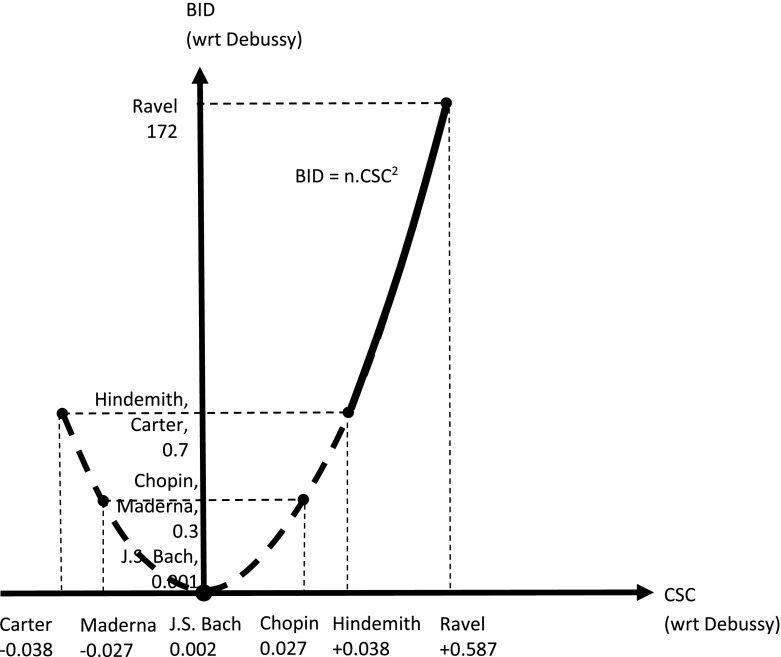
*BID* versus *CSC* rankings. *BID* index as a quadratic function of the *CSC* index. *Source*: Constructed by the author on the basis of results given in Table 4

## Appendix: *BID* versus *CSC* rankings: the *BID* index as a quadratic function of the *CSC* index

The binomial index of dispersion (or similarity) used in Smith and Georges (2014, 2015) can be computed for any pair (*i*, *j*) as:

5 $$BID_{i,j} = {{n(ad - bc)^{2} } \mathord{\left/ {\vphantom {{n(ad - bc)^{2} } {\left[ {(a + b)(c + d)(a + c)(b + d)} \right]}}} \right. \kern-0pt} {\left[ {(a + b)(c + d)(a + c)(b + d)} \right]}},$$

where *a*, *b*, *c*, *d*, and *n* are the count/number of composers in each of the five sets $CI_{i,j}$, $I_{i, - j}$, $I_{j, - i}$, $C - CI_{i,j} - DI_{i,j}$ and *C* (see Table 3). Table 3 permits computation of frequency of joint presence, frequency of joint absences, and frequency of mismatches. When two composers are independent (lack of association), the proportion or frequency of joint influences (*a/n*) is equivalent to the product of the proportions (*a* + *b*)/*n* and (*a* + *c*)/*n* (that is, the proportion of composers in the database that have influenced *i* and the proportion of composers that have influenced *j*). If the observed frequency is greater than the one expected under independence, then the two composers are said to be positively associated. Under the condition that all expected frequencies in the presence/absence table (which is computed assuming independence of composers) are at least five and the sample size is sufficiently large, *BID* is asymptotically $\chi^{2}$ distributed with one degree of freedom. The $\chi^{2}$ test of independence can then be used to assess whether there is a statistically significant association between two composers.

A concrete example is given in Table 4 for composer Debussy. One intriguing point in Table 4 is that the rankings produced by the binomial index and the centralised cosine measure are exactly the same for a large portion of the table but then start to dissociate with composer Carter (identified at the 480th position in the first column of Table 4 and at the 241rd position in the fifth column). This result has, however, a simple explanation—There is a quadratic relationship between *BID* and *CSC*, as proved mathematically in Smith et al. (2015), and *CSC* can take negative values. Observing Eqs. (3) and (5), it is clear that the relationship between *CSC* and *BID* is quadratic:

6 $$BID_{i,j} = nCSC_{i,j}^{2} .$$

Given Eq. (6) and because *CSC* can take negative values, *BID* is not a monotonic function of *CSC*. Hence, the order (or ranking) between *CSC* and *BID* is not preserved over the full set of values for *CSC*.^42^ It is clear for example that a value for *CSC* = +*x* for a pair of composer and *CSC* = –*x* for another pair will generate a unique value *BID* = *nx* ^2^ for both pairs. Hence, the ranking of both pairs of composers will be the same using the *BID* index but quite different with the *CSC* index. This is shown in Fig. 7 using some data given in Table 4. The *CSC* index for Carter and Debussy is −0.038 while the *CSC* for Hindemith and Debussy is +0.039. The *CSC* values for the two pairs of composers are clearly different (one is positive and the other negative, while their absolute value is roughly the same), and the rankings for Carter and Hindemith with respect to Debussy on the basis of *CSC* will therefore be quite distinct, even if these *CSC* values generate the (roughly) same positive value for *BID* (+0.7) according to Eq. (6), implying a (roughly) similar ranking under the *BID*.^43^ Finally, that the rankings produced by *BID* and *CSC* are exactly the same for a large portion of Table 4 is due to *CSC* values not symmetrically distributed between −1 and +1. In fact, the smallest negative value is −0.038 (Carter with respect to Debussy) while the largest positive value is +0.587 (Ravel and Debussy). In this example, as long as *CSC* takes a value larger than 0.038 for a pair of composers, then the rankings given by *BID* and *CSC* will be identical. In other words, the range of values for *CSC* given by [0.038, 0.587] in Table 4 or Fig. 7 will generate the exact same rankings under *CSC* and *BID* because, in this example, *CSC* never takes value over the range [−0.587, −0.038].

## Footnotes

1 The literature is huge, but see for example, Collins (2010), Filippova et al. (2012), Vieira et al. (2012), Kaliakatsos-Papakostas et al. (2010); Mostafa and Billor (2009), de Leon (2002), de Carvalho and Batista (2012), and Fazekas et al. (2010).

2 From outside the music world, an obvious parallel would be the issue of ‘resemblance’ between twins. Even if they appear similar to most observers, to their parents they look distinct. In this example, resemblance is subjective in that it depends on the degree of acquaintance one has with the twins.

3 I conveniently start the History of Western classical music development, in the middle of things, with Guido Monaco da Arezzo (990/992—after 1033), the Italian monk who, according to tradition, is credited with the invention of music staff notation (heighted neumes), making it possible to sing a song one has never heard before. According to Kelly (2015), this is the most radical breakthrough in the history of writing and recording music. The dates used for delimiting periods in Fig. 1 are ‘conventional’ but did not represent some kind of a break at the time. Also, some composers may belong to two different periods as their own style changed over their lifetime. In Fig. 1, they have normally been classified into their most representative period. Figure 1 is a partial outline as many composers, even important ones, are not reported. Furthermore, many styles of classical music, especially since the start of the twentieth century, cannot be represented on a deliberately-narrow time line. Some well-known styles from the twentieth century (and representative composers) are, among others, Futurism (L. Russolo, Antheil, Honegger, Chavez), Micro-Tonal (Haba, Partch, Scelsi), Computer-based (Xenakis, Cage), Electronic and Electro-Acoustic (Varèse, P. Schaeffer, P. Henry, M. Babbitt, M. Davidovsky, Xenakis, Berio, Stockhausen), Aleatory (Cage, Feldman, Boulez, Stockhausen, Berio, Lutoslawski), Minimalism (La Monte Young, Riley, Reich, Glass, J. Adams). For a more detailed structured time line see, for example, Arkhipenko (2015).

4 Transitional composers could represent ‘missing links’ between major periods/styles; they can also be composers whose innovations have generated considerable influences and radical departure from the music of their period (e.g., Debussy or Schoenberg). The ‘transitional’ or ‘innovative’ composers along the diagonal line have been rigorously selected on the basis of composers’ descriptions given in T&G (2013). The period of transition from Baroque to Classical, commonly known as the ‘Pre-Classical’ or Rococo period is, according to T&G (2013), “a historiographical black hole that scholars tried to plug by searching for a ‘missing’ link between the Bach/Handel and Haydn/Mozart poles.” It encompasses both the ‘*Empfindsamer Stil*’ (sensibility style) and the ‘*Style Galant*’, for which CPE Bach and JC Bach, respectively, are representative composers. But it includes several other transitional composers and schools, such as the Milanese school of early symphonists (e.g., G.B. Sammartini—the first big name in the history of the Symphony) that predates the Mannheim’s school (e.g., J. Stamitz) and the first Viennese school (e.g., Haydn), and very influential currents in opera (e.g., the opera seria of Metastasio’s composers Vinci and Hasse, the dramma giocoso/opera buffa of Galuppi and Pergolesi, and the opera reforms of Gluck and Jommelli). The case for putting de Muris and de Vitry on the diagonal is perhaps worth mentioning. They were both mathematicians and musicians. According to T&G (2013), their treaties and the debates they sparkled, and their notational breakthroughs and innovations had enormous repercussions. “So decisive were their contributions that this theoretical tradition has lent its name to an entire era”—Ars Nova (which is also the title of the Treatise of de Vitry).

5 The title of the book can be translated as: “History of Music in Italy, Germany and France—From the Earliest Christian Times to the Present. See: https://archive.org/stream/geschichtedermus01bren#page/n5/mode/2up.

6 This kind of approach is conceptually not much different from understandings of biology and phylogeny through the study of genetics, whereby one identifies the lineages and the way population splits over time into new species.

7 Defining the ‘meme’ replicator as a unit of cultural transmission or a unit of imitation, Dawkins (1976) suggests that “[j]ust as genes propagate themselves in the gene pool by leaping from body to body via sperms or eggs, so memes propagate themselves in the meme pool by leaping from brain to brain via a process which, in the broad sense, can be called imitation.”

8 If a music meme is to dominate the attention of a composer brain, it must do so at the expense of rival memes, hence the competition or differential fitness factor.

9 An “Appendix” to this section also discusses why the centralised cosine measure as a statistical measure of association is, in our opinion, a better measure than the binomial index of dispersion for the project at hand.

10 See Smith and Georges (2014) for a review of the philosophy and methodology underlying the *CMN*.

11 This is an approach reminiscent of the approaches used in biodiversity analyses of observed diversities (in a population or group of populations of organisms) and distributions between given areas, to identify relational patterns useful to explaining the historical evolution of the forms under study. See Cheetham and Hazel (1969) and Hayek (1994) for good surveys of related works and methods such as the “measures of association” (also named in a somewhat interchangeable way “similarity,” “resemblance,” or “matching” indices).

12 Table 1 provides the full name of the ecological characteristics of the three composers.

13 A common illustration of this convergent evolution is the parallel evolution taking place in Australian marsupials versus placental mammals elsewhere.

14 Dozens of measures of association have been studied in the biosystematics literature, such as the first and second Kulczynski coefficients (1927), the Jaccard coefficient (1901), the Dice coefficient (1945), the Simpson coefficient (1943), the binary distance coefficient (Sneath 1968), the binomial index of dispersion $\chi^{2}$ statistic (Potthoff and Whittinghill 1966), the Salton’s measure (1987) or its equivalent, the cosine similarity measure discussed in scientometrics and bibliographic coupling literature (Sen and Gan 1983; Glänzel and Czerwon 1996).

15 The “Appendix” provides a comparison between the centralised cosine measure and another well-known measure, the binomial index of dispersion, that was used in Smith and Georges (2014, 2015).

16 Note that square root of 1 is ±1, which is why we take the absolute value, *ABS*.

17 Table 4 indicates that we can reject the null hypothesis of no association between Debussy and the first 181 composers of the table (until and including Monk in Table 4) at a 5% significance level. Table 4 also shows results for the binomial index of dispersion discussed in the “Appendix”. Note that as shown in last column of the table, the binomial statistic for Debussy and Ravel is 172.1. Using the $\chi^{2}$ distribution, the critical value at a 5% significance level is 3.84. (For significance levels at 1 or 10%, the critical values are 6.63 and 2.70, respectively.) Because 172.1 > 3.84, we reject the null hypothesis of no association between Debussy and Ravel in favor of the alternative that these two composers are statistically significantly associated (in agreement with the conclusion drawn from the CSC index). Observe that we can reject the null hypothesis of no association with Debussy for the first 181 composers of the table: this is the same cut-off point for both the $\chi^{2}$ test (binomial index) and the Z-statistic (CSC index).

18 In the case of Carter and Debussy, the centralised cosine value of −0.038 is not far enough from zero to be statistically significantly negative. The Z-statistic reported in Table 4 is 0.844 which is lower than the critical value of 1.96 at a 5% significance level under the standard normal distribution. We therefore cannot reject the null hypothesis of no association between Debussy and Carter.

19 Figure 4 reports the top 10 most similar composers to J. Haydn, W. A. Mozart, and Schubert. It does not report the similarity index between J. Haydn and Schubert as Schubert is the 19th most similar composer to J. Haydn. Figure 4 also reports that Mozart, Beethoven, Gluck and J. C. Bach are the four most similar composers to J. Haydn. Haydn, Boccherini, J. C. Bach, and Salieri are the four most similar composers to Mozart. Reicha, Mendelssohn-Hensel, Salieri, and Taneyev are the four most similar composers to Schubert.

20 Figures 4 and 5 also give the birth date of each composer (in front of the name) so that we can compute the sum of the age differentials between all composers similar to Schubert and Schubert himself. The sum is −49 years in the influences network case, and +161 years in the ecological database case. This clearly indicates that while the personal influences network associates Schubert with composers relatively older than him, the ecological database associates him with much younger composers. The same calculations for Mozart provide sums of age differentials of −40 years and −145 years, respectively, demonstrating that the ecological niche of Mozart was rather backward-looking. For Haydn, we get +148 years and +43 years, respectively. Although Haydn’s musical ecological niche is clearly forward-looking (as he is typically associated with innovations in Symphonic and String Quartets compositions), Haydn is also forward-looking with respect to his influences network. From this perspective, his ecological niche is in concordance with his influences network, as in the case of Mozart. This is not the case for Schubert.

21 Although we do have negative associations between composers, none of them are statistically significant.

22 Using Beethoven as an example of how Table 5a is constructed on the basis of Fig. 6, observe that panels 3 and 4 in Table 5a show that 33% of Beethoven’s younger contemporary composers are located in the North-East quadrant of Fig. 6h while only 6% of composers born 0 to 25 years after Beethoven’s death are located in the North-East quadrant of Fig. 6a. In Fig. 6h, we see indeed that 18 ‘object’ composers are in the North-East quadrant out of a total of 54 composers included in the graph. Setting *y* = 18 and *n* = 54, we get that *p* = 18/54 = 0.33 as reported in Table 5a. For Fig. 6a, *y* = 2, *n* = 33, and *p* = 0.06. We can test whether the difference in the two proportions *p*1 and *p*2 is statistically different, that is: *H*_0_: *p*1 = *p*2 versus *H*_*A*_: *p*1 ≠ *p*2. We need to compute: $Z^{*} = (p1 - p2)/\sqrt {p^{*} (1 - p^{*} )\left( {\frac{1}{n1} + \frac{1}{n2}} \right)}$ where $p^{*} = \frac{y1 + y2}{n1 + n2}$. In our example, $Z^{*} = 2.94 > 1.96$. Hence we reject the null hypothesis that the two proportions *p*1 and *p*2, are the same, in favour of the alternative that their difference is statistically significant at a 5% level. Alternatively, in terms of *P* value, Pr(*Z* > 2.94) = 0.0016 < 0.05, and we again reject the null hypothesis.

23 This is also confirmed when looking at the first column in panel 4 of Table 5b (representing ‘subject’ composers vs. composers born 0–25 years after the death of the ‘subject’ composers). Only 4% of the composers are strongly similar to the (dead) ‘subject’ composers, while 61% are statistically independent across both personal and ecological categories.

24 Composers who belong to the ‘canon’ of classical music are not necessarily ranked, but scientists can count the number of lines or pages devoted to them in major music encyclopedia, the number of recordings available, etc., and then turn scores into a ranking. Although the rankings *per se* (and underlying aggregation methodology) are controversial and often discredited by musicologists, the collection of names in these lists, instead of the ranking, may provide useful information.

25 I left out the group of pre-Renaissance composers. Also, whether a composer falls into a specific period is based on the categories given in the *CMN*.

26 Smith’s ‘primary’ ranking for Top-20 composers is as follows: (1) Bach, JS; (2) Mozart, WA; (3) Beethoven; (4) Schubert; (5) Brahms; (6) Wagner; (7) Verdi; (8) Handel; (9) Haydn, J; (10) Chopin; (11) Tchaikovsky; (12) Liszt; (13) Schumann, R; (14) Debussy; (15) Puccini; (16) Stravinsky; (17) Mendelssohn; (18) Strauss, R; (19) Mahler; and (20) Ravel. Smith’s primary ranking is based on scores received by these composers on a combination of eleven (unweighted) variables such as the length of composer entry in the *Grove's Dictionary of Music*, the total number of recordings referring to each composer, etc. See Smith (2000).

27 Smith computes a weighted index for the diffusion of the influence of a composer *i* based on three elements: (1) the list of all composers *k* influenced by *i*. (2) The primary ranking (referred above) of those composers *k*, *PRK*_*k*_, and (3) A function *f* associating a greater weight *W*_*k*_ to the names of better ranked composers. According to Smith’s methodology, the Top-20 most influential composers are: (1) Bach, JS; (2) Debussy; (3) Wagner; (4) Stravinsky; (5) Mozart; (6) Beethoven; (7) Liszt; (8) Chopin; (9) Schumann; (10) Schoenberg; (11) Ravel; (12) Bartok; (13) Brahms; (14) Mendelssohn; (15) Strauss, R.; (16) Berlioz; (17) Handel; (18) Haydn, J; (19) Palestrina; and (20) Rossini.

28 Murray’s top 20 composers are: (1) Beethoven; (2) (tied) Mozart; (3) Bach, JS; (4) Wagner; (5) Haydn, J; (6) Handel; (7) Stravinsky; (8) Debussy; (9) Liszt; (10) Schubert; (11) Schumann; (12) Berlioz; (13) Schoenberg; (14) Brahms; (15) Chopin; (16) Monteverdi; (17) Verdi; (18) Mendelssohn; (19) Weber; and (20) Gluck.

29 On a cautious note, the difference between the two proportions is not statistically significant according to the methodology presented in Footnote 22. Hence we should avoid extracting too much musicological information from this fact.

30 For example, much of the traditional symphonic writing fell out of fashion after Beethoven’s Ninth Symphony (1824). That Joachim Raff (1822–1882), a prolific and very well-known traditional symphonist of his time (but born just 5 years before the death of Beethoven), tends to get little attention in music history books, shows the ease with which the historian’s attention is captured by novelty.

31 We can test the difference in proportions using the methodology in Footnote 22. The differences are statistically significant at 5% for Monteverdi, Gluck, and Beethoven versus their corresponding ‘average’ composers of the period from which they progressively diverged. For Debussy and Schoenberg versus the ‘average’ Romantic composer, however, we cannot reject the null hypothesis of no difference in proportions.

32 As evidenced by a letter from 1923 to conductor Werner Reinhart, reproduced and translated in Stein (1975) in his selected writings of Schoenberg, the composer wrote: “I do not attach so much importance to being a musical bogeyman as to being a natural continuer of properly-understood good old tradition!”.

33 As T&G (2013) recount, the pianist and conductor Hans von Bülow hailed it as the ‘Tenth Symphony’ and then proclaimed a new holy trinity of classical music—‘Bach, Beethoven and Brahms’—that has lived on, ever since, in the catchphrase ‘the three B’s’.

34 As T&G (2013) explain, A. Scarlatti, a culminating figure of Baroque opera at the turn of the 18^th^ century, laid the foundation of *opera seria* (serious opera) and the *da capo* aria which includes a last section that is essentially unwritten but becomes an opportunity for the singer to do free-form spontaneous embellishment and improvisation, ensuring a virtuoso display and the kind of spectacular performance on which public opera has always thrived. With time, most great singers (of which the well-known Farinelli) carried around a portfolio aria that could be inserted whenever they sang, even if irrelevant to the context. Although the operas of Handel are cast in the same mold as other *opera seria*, he typically gave performers less room to manoeuvre. This led to a decline in interests from performers and the public and forced Handel out of opera and into English oratorios.

35 Incidentally, the relative position of Rameau and Pergolesi in this graph reminds the so-called ‘*Querelle des bouffons*’ (the War of the Buffoons), which in the 1750s (a long generation before the French revolution) foreshadowed not only musical change but also political and social change. As T&G (2013) explain, Jean-Jacques Rousseau and Diderot ridiculed the high-minded French *Tragédie Lyrique* and *Pastorale Héroique* in the style of Lully, Rameau, Leclair (*Scylla et Glaucus*), and Mondonville (*Titon et l’Aurore*), which was performed by the royal musical establishment. Furthermore, Rousseau argued that French language was not suitable for operas. Instead, Rousseau was glorifying the ‘modern’ style of Italian *operas buffa* and *intermezzos*, including the most popular at the time—*La serva padrona*—by Pergolesi, brought to Paris in 1752.

36 Liszt’s teacher, Carl Czerny, was himself the pupil of Beethoven (among others). Czerny’s compositional style and teaching often mimicked Beethoven himself, and much of Franz Liszt’s early learning can be said to have come from Beethoven himself (Mao 2012).

37 Quoting the Hegelian historian of music Franz Brendel, cited in T&G (2013), “[Liszt’s] symphonic poems was a new synthesis, a transcendence giving rise to a new thesis—a new dawn—for music (…). It is the unity of the poetic and the musical, and the progress to a new consciousness of this unity, that deserves to be called the essential novelty in the artistic creations under discussion.” Interestingly, when comparing the respective contribution of Liszt and Brahms in the history of music development, T&G (2013) argue that Brahms’s first symphony (composed in 1876), “was crucial in making the traditional symphony a viable option once again, a genre that could now be pursued without the stigma of being considered unoriginal and unimaginative.” Therefore, they add, “[t]here would henceforth be two interpretations of the great tradition: the radical historicist one of Liszt and Wagner, which cast it as a kind of permanent revolution, and Brahms’s liberal evolutionist one, which cast it as an incremental and consensual growth, building on a gloried past.”

38 In the words of musicologist Deryck Cooke (1980), “Bruckner created a new and monumental type of symphonic organism, which abjured the tense, dynamic continuity of Beethoven, and the broad, fluid continuity of Wagner, in order to express something profoundly different from either composer, something elemental and metaphysical.”

39 This French spirit in music was summed up in the slogan *Ars Gallica*—‘French Art’, but, according to T&G (2013), “when applied to a certain school of composers, it meant a French art that arose in nationalistic response to losing the Franco-Prussian War of 1870–71.”

40
*Les Six* is a name given in 1920 to a group of six French composers who worked in Paris, of which Milhaud, Poulenc and Honegger are the most well-known.

41 The famous rivalry between ‘Piccinnists’ and ‘Gluckists’, a ‘querelle’ in Paris opposing dramatic versus musical values of two operas composed on the same subject, *Iphigénie en Tauride*, seems to suggest a stronger differentiation than the one implied by the North-West location of Piccinni versus Gluck. However, for T&G (2013) “[b]oth were equally, though differently, a sign of the intellectual, philosophical, and social changes that were taking place over the course of the eighteen century. (…) The two composers, privately on friendly terms, were more allies than rivals,” suggesting indeed a convergent evolution despite their respective advocates and their conflicting network of influences that led them to a collision course.
